# Claudin-7 deficiency induces metabolic reprogramming of neutrophils in the colorectal cancer microenvironment

**DOI:** 10.1038/s41419-025-08064-3

**Published:** 2025-10-16

**Authors:** Xiaoqing Liang, Dajin Yuan, Songyun Zhao, Jianbo Zhou, Kun Wang, Xiaoli Liu, Yin Liu, Huimin Li, Mengdi Hao, Wenbin Huang, Wenjie Li, Lei Ding

**Affiliations:** 1 Department of Gastrointestinal Surgery, Beijing Shijitan Hospital, Capital Medical University, Beijing, China; 2 Department of Plastic Surgery, The First Affiliated Hospital of Wenzhou Medical University, Wenzhou, China; 3 Department of Thoracic Surgery and Institute of Thoracic Oncology, Frontiers Science Center for Disease-Related Molecular Network, West China Hospital, Sichuan University, Chengdu, China; 4 Department of Ultrasound, Beijing Friendship Hospital, Capital Medical University, Beijing, China

**Keywords:** Immune evasion, Gastrointestinal cancer

## Abstract

Neutrophils are integral components of the bone marrow and stromal cell network. They express the immune checkpoint molecule PD-L1 and can induce T cell exhaustion, thereby promoting immunosuppression. In this study, we investigated whether tumor-derived *Cldn7* deficiency could recruit polymorphonuclear neutrophils (PMNs), induce their metabolic reprogramming, and consequently drive their transition toward a pro-tumor phenotype, leading to the establishment of an immunosuppressive tumor microenvironment (TME). Using single-cell RNA sequencing, clinical sample validation, and both in vivo and in vitro experiments, we found that *Cldn7* deficiency in colorectal cancer (CRC) results in a tumor microenvironment characterized by significantly increased infiltration of CD11b⁺ Ly6G⁺ neutrophils. This is accompanied by neutrophil metabolic reprogramming that facilitates their phenotypic shift toward a tumor-promoting state, which in turn suppresses the cytotoxic function of CD8⁺ T cells and contributes to the formation of an immunosuppressive TME, thereby accelerating CRC progression. Mechanistically, *Cldn7* deficiency indirectly activates the NF-κB signaling pathway, leading to elevated secretion of chemokines such as CXCL1 that are responsible for PMN recruitment. Inhibition of the NF-κB/CXCL1 axis reduces PMN infiltration, decreases PD-L1 expression on neutrophils, suppresses neutrophil glycolysis and histone lactylation, reverses the exhausted phenotype of CD8⁺ T cells, thereby mitigating the immunosuppressive microenvironment. Furthermore, overexpression of *Cldn7* enhances the efficacy of immune checkpoint blockade (ICB) therapy.Collectively, our findings indicate that *Cldn7* deficiency not only contributes to immune evasion and malignant progression in CRC but also plays a critical role in immune modulation. Targeting PMN metabolic reprogramming and immunosuppressive function associated with *Cldn7* loss may offer a promising strategy to improve the therapeutic efficacy of immunotherapy in CRC.

## Introduction

Colorectal cancer (CRC) is one of the most common malignant tumors of the digestive system, ranking third globally in incidence and leading in mortality, posing a severe threat to human health. The development of CRC is a multi-stage, complex biological process involving various factors contributing to its progression and malignant transformation [1–3]. Current treatments for CRC include traditional surgery, radiotherapy, chemotherapy, and emerging biological immunotherapies [4]. Immune checkpoint inhibitor therapy (ICT) for CRC has been challenging to achieve effective results due to the formation of an immunosuppressive tumor microenvironment (TME) [5, 6]. Therefore, studying the molecular mechanisms underlying the formation of the immunosuppressive microenvironment in CRC may provide a theoretical basis for improving ICT treatment for CRC.

Cldn7 is a member of the Claudins family of tight junction proteins, expressed in the apical and lateral membranes of intestinal epithelial cells, as well as in the basal crypt stem cell regions of the intestine [7, 8]. Studies have shown that, in addition to its classic tight junction functions such as barrier maintenance and cell polarity, which are crucial for the physiological functions of various organs, Cldn7 also plays significant roles in the initiation of inflammation and tumorigenesis [9, 10]. Downregulation of Cldn7 expression has been observed in both inflammatory and tumor tissues. Our data indicate that low expression of *Cldn7* in tumor tissues is a biomarker for poor prognosis in CRC patients. Previous research has also confirmed that *Cldn7* deletion significantly affects the inflammatory microenvironment of tumors in mice, primarily through changes in neutrophil infiltration. However, the specific mechanisms remain unclear and require further investigation.

The tumor microenvironment (TME) consists of various cells and extracellular components, including endothelial cells, fibroblasts, immune cells, and extracellular components such as cytokines, growth factors, hormones, and extracellular matrix [11, 12]. These elements surround tumor cells, are nourished by an intricate network of microvessels, and form a localized biological microenvironment that influences tumor progression [13–15]. The role of tumor-associated polymorphonuclear neutrophils (PMNs) in tumor development has been underexplored. Recent studies have gradually revealed the role of PMNs in tumorigenesis and progression [16, 17]. Despite the diversity of their functions, a high density of PMNs in tumor tissue is usually associated with a poor prognosis. Under the influence of the tumor microenvironment, these cells are induced to transform into subtypes with immunosuppressive features, which suppress the body’s immune response to the tumor and thus contribute to the spread of cancer cells and their resistance to immunotherapy [18, 19]. However, the specific molecular mechanisms driving neutrophil phenotypic changes have not been fully elucidated. PMNs can secrete cytokines or form neutrophil extracellular traps (NETs) to regulate tumor metastasis [20]. At the primary tumor site, PMNs promote tumor cell migration and invasion by secreting cytokines or directly interacting with tumor cells [21, 22]. Research indicates that PMNs play a critical regulatory role in the pathological processes of tumor initiation, progression, invasion, and metastasis [23, 24]. Additionally, clinical studies have reported that the neutrophil/lymphocyte ratio in peripheral blood is an independent prognostic factor for CRC patients [25, 26]. However, due to the phenotypic diversity and highly heterogeneous functions of PMNs, their biological role in the malignant progression of CRC remains to be fully elucidated.

Neutrophils are part of the bone marrow and stromal cell network, express PDL1, and drive immune checkpoint engagement and T cell exhaustion [27]. In this study, we investigated whether tumor-derived *Cldn7* deletion attracts PMNs infiltration and promotes their shift to a pro-tumorigenic phenotype, resulting in an immunosuppressive microenvironment. Using single-cell sequencing, clinical sample validation, and in vivo and in vitro experiments, we found that *Cldn7* deletion leads to increased recruitment of CD11b^+^Ly6G^+^ neutrophils in the CRC microenvironment and accelerates malignant progression of CRC.*Cldn7* deletion indirectly activates the NF-kB signaling pathway, leading to increased secretion of PMN-recruitment-associated chemokines (e.g. CXCL1). Furthermore, deletion of *Cldn7* expression in the CRC microenvironment leads to adaptive metabolic reprogramming of PMNs, as evidenced by elevated glycolysis mediated by histone lactylation, a higher proportion of PDL1^+^ PMNs expression, and an antiapoptotic phenotype of neutrophils (increased expression of the *Bcl2*). Inhibition of the NF-kB/CXCL1 axis in *Cldn7*-deficient tumor cells ameliorates the immunosuppressive microenvironment by decreasing PMN recruitment, decreasing PD-L1 expression on PMNs, and reversing the CD8^+^ T cell exhaustion phenotype. In addition, overexpression of *Cldn7* improved the efficacy of ICB therapy. In conclusion, our experimental results suggest that *Cldn7* deficiency plays an initiating role in the malignant progression of CRC and has an immunomodulatory function, and that the study of related biological processes may improve the efficacy of CRC immunotherapy in the future.

## Results

### Single-cell analysis reveals a potential regulatory role of tumor-derived *Cldn7* in polymorphonuclear neutrophils (PMNs) within colorectal cancer

**Fig. 1:**
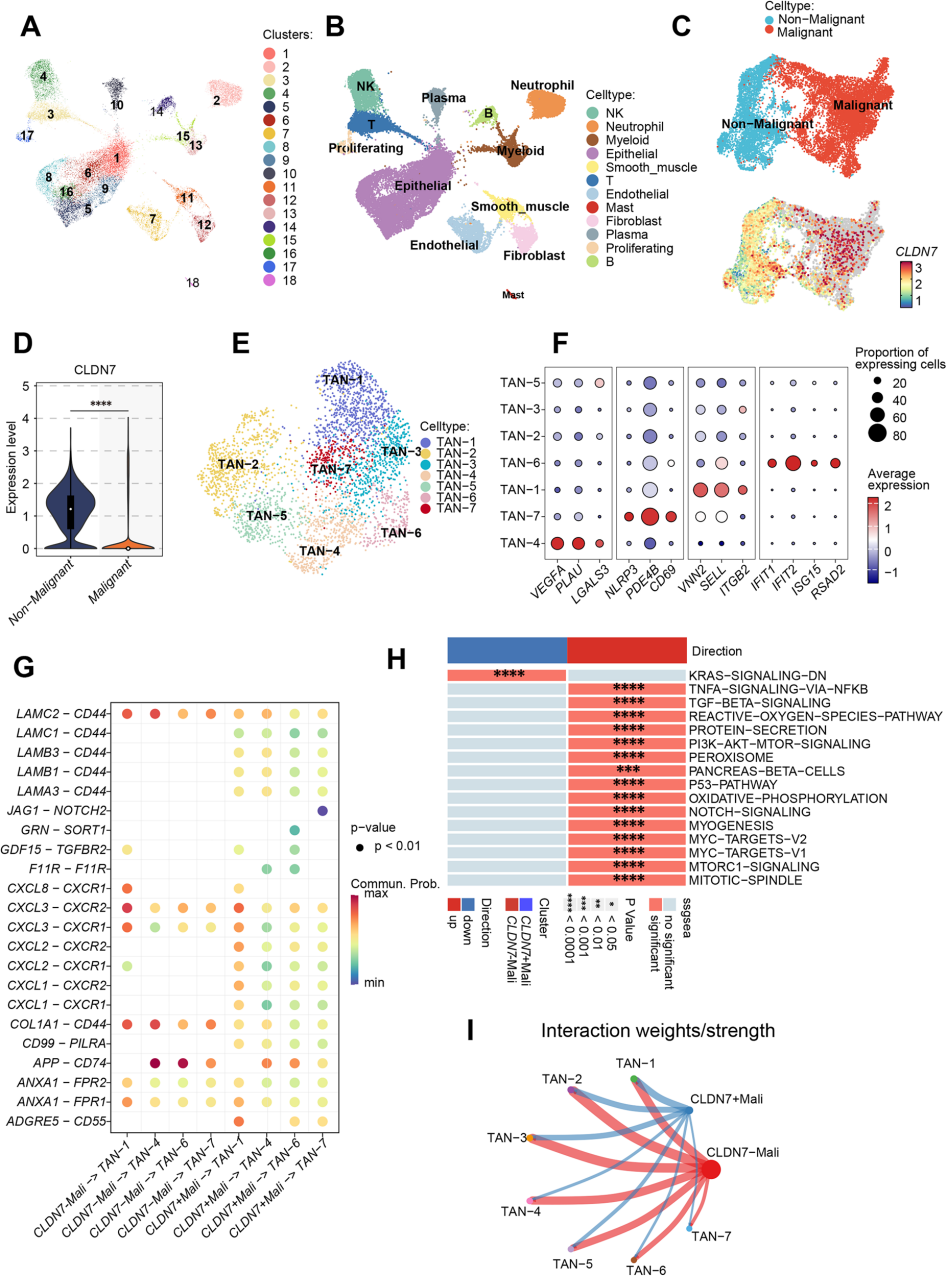
Single-cell sequencing data of colorectal cancer illustrates the negative correlation between tumor suppressor gene *Cldn7* expression levels and neutrophil infiltration within the tumor microenvironment. **A** UMAP clustering of single cells showing distinct cell clusters labeled 1–18. **B** Cell-type annotation based on clusters, identifying various cell types including NK cells, neutrophils, plasma cells, proliferating cells, epithelial cells, smooth muscle cells, endothelial cells, fibroblasts, and mast cells. **C** UMAP plot displaying malignant (red) versus non-malignant (blue) cell populations, with *CLDN7* expression levels indicated by a color gradient. **D** Violin plot comparing *CLDN7* expression levels between non-malignant and malignant cells, showing significantly lower expression in malignant cells. **E** UMAP plot identifying subclusters of tumor-associated neutrophils (TANs), labeled TAN-1 through TAN-7. **F** Dot plot showing the expression of selected genes across TAN subclusters, with dot size representing the proportion of expressing cells and color indicating average expression levels. **G** Dot plot of ligand-receptor interactions between *CLDN7*-expressing cells and TAN subclusters, with color indicating maximum communication probability and significant interactions marked. **H** Pathway enrichment analysis of genes associated with *CLDN7* and TAN subclusters, highlighting significantly enriched pathways and their direction (upregulated or downregulated). **I** Network diagram showing the interaction weights and strengths among TAN subclusters, with red and blue lines representing stronger or weaker interactions, respectively.

**Fig. 2:**
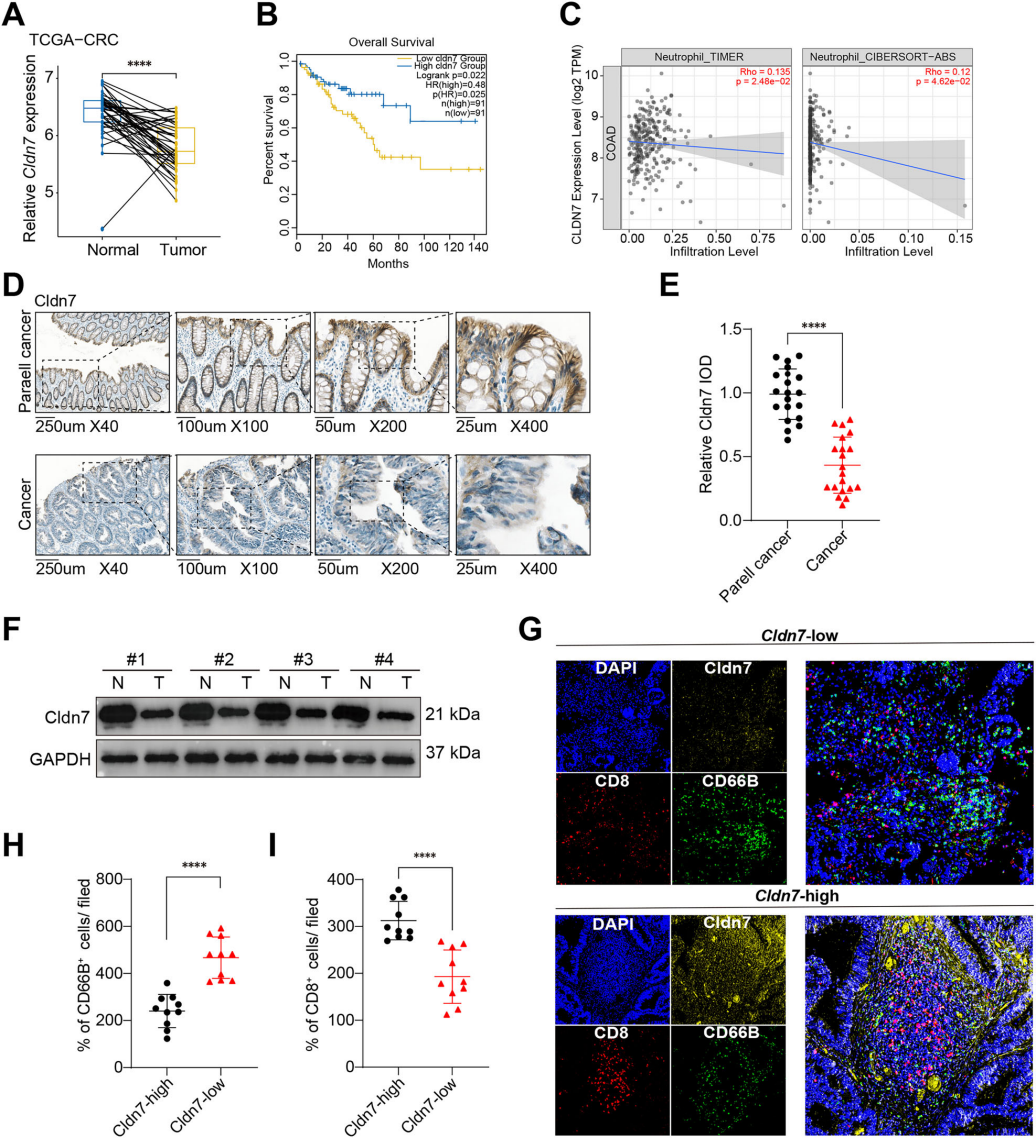
*Cldn7* is expressed at low levels in CRC tumor tissues and is negatively correlated with PMN infiltration. **A** Comparison of *CLDN7* mRNA expression levels in normal colorectal tissues and colorectal cancer tissues from TCGA-CRC data, showing a significant reduction in cancer tissues (****p < 0.0001). **B** Kaplan–Meier survival analysis showing a significantly worse overall survival (OS) in colorectal cancer patients with low *CLDN7* expression compared to those with high *CLDN7* expression (*p* = 0.024). **C** Correlation between *CLDN7* expression (log2 TPM) and neutrophil infiltration levels using TIMER and CIBERSORT-ABS methods. Negative correlations were observed in both models, where the infiltration level increases as CLDN7 expression decreases. **D**, **E** Immunohistochemical staining of *CLDN7* in normal (upper panel) and colorectal cancer tissues (lower panel) at different magnifications, showing that *CLDN7* is primarily localized in the epithelial cells in normal tissue and is significantly reduced in cancer tissues (****p < 0.0001). **F** Western blot analysis of *CLDN7* expression in colorectal cancer patient samples from tumor and adjacent normal tissues, the image suggests that *CLDN7* expression is reduced in CRC tumor tissues compared to the adjacent normal tissues. **G**–**I** Immunofluorescence staining of colorectal cancer tissues showing immune cell infiltration in low (upper panel) and high (lower panel) *CLDN7* expression tissues. Tissues with low *CLDN7* expression exhibit fewer CD8-positive T cells (red) and more CD66B-positive neutrophils (green), while high *CLDN7* expression associated with significantly reduced neutrophil infiltration and increased CD8-positive T cell infiltration (****p < 0.0001).

Claudin7 (Cldn7) has previously been recognized as a tumor suppressor gene, yet its specific functional mechanisms in colorectal cancer (CRC) progression remain largely unclear [10, 28]. To systematically investigate the biological role of Cldn7, we performed single-cell RNA sequencing on tumor samples from five clinically diagnosed CRC patients. Using UMAP-based clustering analysis, we classified and annotated 12 major cell types, including NK cells, neutrophils, epithelial cells, myeloid cells, smooth muscle cells, T cells, endothelial cells, antigen-presenting cells, plasma cells, proliferating cells, and B cells (Fig. 1A, B, Supplementary Fig. 1A, B). Subsequently, we conducted a more detailed analysis of the epithelial cells using inferCNV, identifying 14 epithelial cell subpopulations and obtaining inferCNV scores for each (Supplementary Fig. 1C, D). Based on these scores, we classified epithelial subpopulations in the CRC single-cell dataset into malignant and non-malignant epithelial cells and compared Cldn7 expression levels between these two groups. The results showed that Cldn7 was significantly downregulated in malignant epithelial cells, with some malignant CRC epithelial subpopulations exhibiting a complete loss of Cldn7 expression (Fig. 1C, D). In further analysis, we also observed the heterogeneity of neutrophils in the CRC microenvironment. Through subpopulation clustering, we identified seven distinct tumor-associated neutrophil (TAN) subgroups (Fig. 1E) [29]. Among the seven TAN subpopulations identified in CRC, TAN-1 exhibited high expression of genes related to neutrophil transendothelial migration, such as VNN2, SELL, and ITGB2, suggesting that this cluster may represent a transitional state from PMNs to TANs. TAN-4 was characterized as a pro-tumorigenic subpopulation, showing elevated expression of multiple pro-angiogenic and pro-metastatic factors, including VEGFA, PLAU, and LGALS3. This subset also displayed a distinct transcriptional profile, with upregulation of a series of interferon-stimulated genes (ISGs), such as IFIT1, IFIT2, IFIT3, ISG15, and RSAD2 (Fig. 2F). TAN-7 belonged to an inflammation-related subcluster, with strong expression of inflammatory regulators NLRP3 and PDE4B, along with high levels of the neutrophil activation marker CD69. Additionally, TAN-6 demonstrated marked expression of tumor-promoting molecules, including IL1RN and adrenomedullin (ADM) (Fig. 2D), indicating its potential role in modulating the tumor microenvironment. Subsequently, ligand–receptor interaction analysis revealed intercellular communication differences between malignant cells with high and low Cldn7 expression and different TAN subpopulations. Notably, several chemokine pathways—including CXCL1–CXCR1, CXCL2–CXCR1, and CXCL8–CXCR1—were significantly involved, suggesting that Cldn7 may play a role in regulating neutrophil recruitment and function (Fig. 1G). Furthermore, hallmark enrichment analysis indicated distinct signaling pathway enrichments in CRC malignant epithelial cells with high versus low Cldn7 expression. Low Cldn7 expression was potentially associated with enrichment in signaling pathways such as TNFα-signaling-via-NFκB, PI3K-AKT-mTOR-signaling, and the p53-pathway (Fig. 1H). Cell interaction analysis also indicated a strong interaction between Cldn7-low malignant epithelial cell subpopulations and TAN subgroups (Fig. 1I). These findings suggest that Cldn7 expression is significantly downregulated in malignant epithelial cells in colorectal cancer and that it may play a key role in reshaping the tumor microenvironment and promoting CRC progression by affecting neutrophil heterogeneity and functional state through aberrant communication networks with TANs.

### *Cldn7* deletion constructs an immunosuppressive microenvironment in CRC in a PMN-dependent manner

**Fig. 3:**
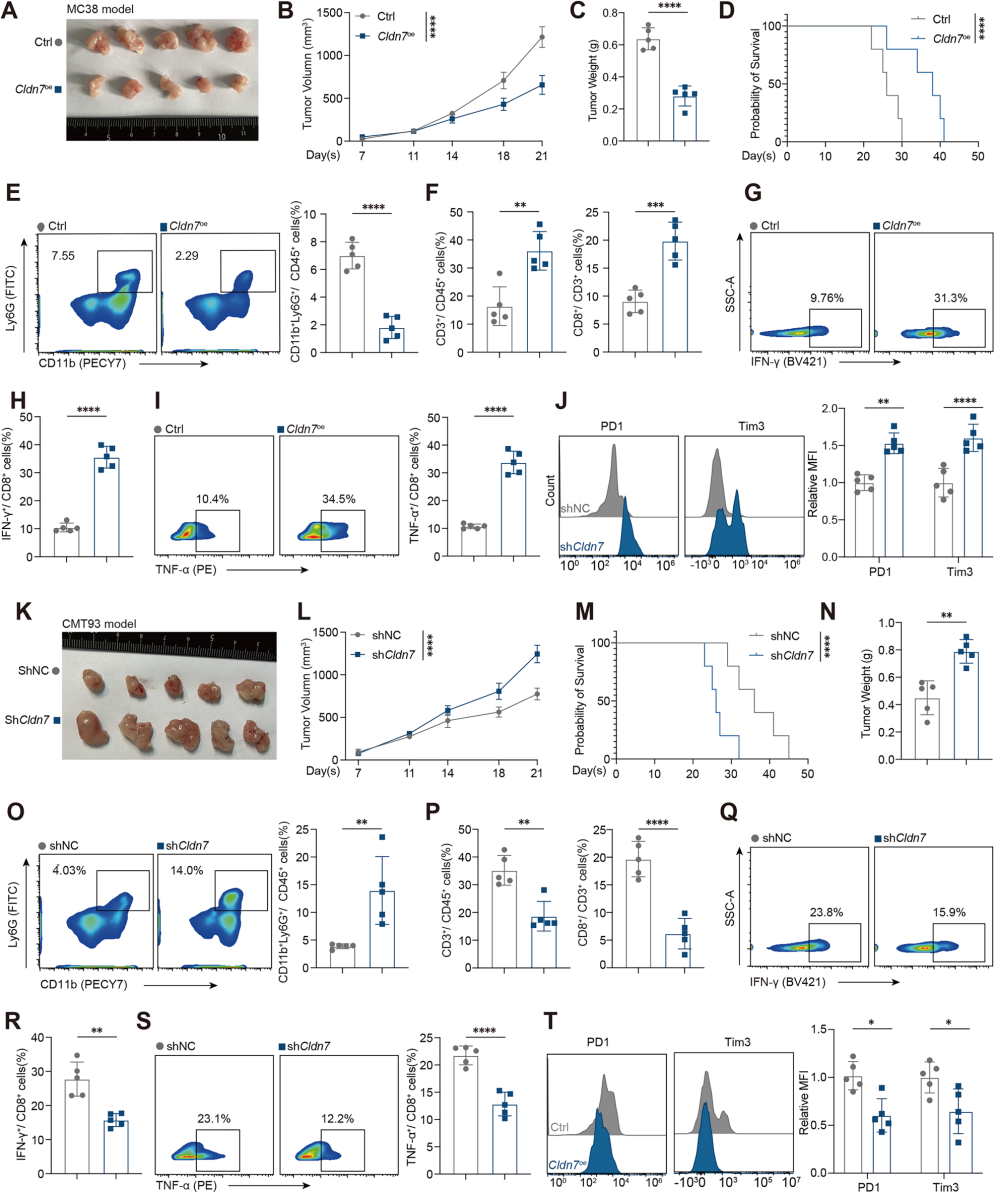
*Cldn7* deficiency promotes neutrophil accumulation in the tumor microenvironment of tumor-bearing mice. (**A**–**D**) In the MC38 tumor model, Cldn7 overexpression (Cldn7⁺⁺) significantly reduced tumor volume (**B**), tumor weight (**C**), and prolonged mouse survival (**D**) compared to the control group (Ctrl); **A** shows representative tumor images. (*n* = 5 per group). Flow cytometry analysis shows that Cldn7 overexpression significantly decreased the proportion of CD11b⁺ Ly6G⁺ neutrophils in the tumor tissue (**E**), while increasing the infiltration of CD8⁺ T cells (**F**). Further analysis revealed that Cldn7 overexpression significantly increased IFN-γ (**G**, **H**) and TNF-α (**I**) production by CD8⁺ T cells. **J** Inhibitory receptor analysis shows that Cldn7 overexpression reduces the expression of PD-1 and Tim-3 on CD8⁺ T cells, suggested reduced T cell exhaustion. **K**–**N** In the CMT93 model, Cldn7 knockdown (shCldn7) promotes tumor growth (**L**), increases tumor weight (**N**), and significantly shortens mouse survival (**M**); **K** shows representative tumor images. Cldn7 knockdown leads to an increased proportion of CD11b⁺ Ly6G⁺ neutrophils in the tumor tissue (**O**), while decreasing CD8⁺ T cell infiltration (**P**). Compared to the shNC control group, shCldn7 significantly reduces IFN-γ (**Q**, **R**) and TNF-α (**S**) production by CD8⁺ T cells. **T** Inhibitory receptor analysis shows that Cldn7 knockdown increases PD-1 and Tim-3 expression levels, suggesting enhanced T cell exhaustion. Data are presented as mean ± SEM, with statistical significance indicated as follows: *p < 0.05, **p < 0.01, ***p < 0.001, ****p < 0.0001.

**Fig. 4:**
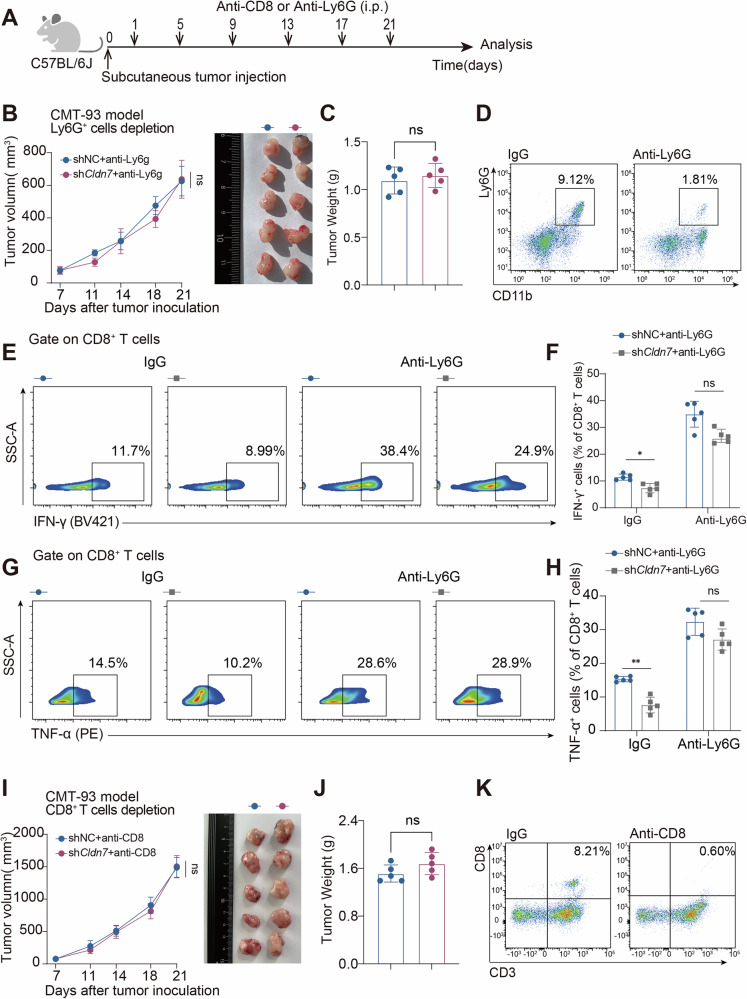
Neutrophil depletion partially restores CD8^+^ T cell function and inhibits tumor progression in *CLDN7*-knockdown tumors. **A** Schematic of the experimental timeline. C57BL/6J mice (*n* = 5 per group) bearing subcutaneous CMT93 tumors (transfected with shNC or shCldn7) were treated intraperitoneally with anti-CD8 or anti-Ly6G antibodies every 4 days starting on day 5 post-inoculation. **B** Tumor growth curves showing that Ly6G⁺ neutrophil depletion significantly reduced tumor volume in the shCldn7 group. Representative tumor images are shown on the right. **C** Tumor weights on day 21; no significant difference was observed between groups. **D** Flow cytometry plots validating effective depletion of CD11b⁺Ly6G⁺ neutrophils after anti-Ly6G antibody treatment. **E**, **F** Representative flow cytometry plots and quantification of IFN-γ⁺CD8⁺ T cells. Anti-Ly6G treatment significantly increased the frequency of IFN-γ⁺ CD8⁺ T cells in the shCldn7 group. **G**, **H** Representative plots and quantification of TNF-α⁺CD8⁺ T cells. shCldn7 + anti-Ly6G tumors showed restored TNF-α production comparable to controls. **I** Tumor growth curves after CD8⁺ T cell depletion showing no significant difference between shNC and shCldn7 groups. Representative tumor images are shown. **J** Tumor weights on day 21 after CD8⁺ T cell depletion; no significant difference. **K** Flow cytometry confirming efficient depletion of CD3⁺CD8⁺ T cells by anti-CD8 antibody. Data are presented as mean ± SEM (*n* = 5 mice per group). Tumor growth curves were analyzed using two-way ANOVA with Bonferroni post hoc test. Tumor weight and flow cytometry data were analyzed using an unpaired two-tailed Student’s *t* test. *P* < 0.05 was considered statistically significant. ns， not significant; *p* < 0.05; *p* < 0.01.

We first performed paired transcriptomic analysis using the TCGA-COAD dataset and observed a significant downregulation of *Cldn7* expression in CRC tumor tissues (Fig. 2A). This finding was further validated by immunohistochemistry and Western blot analysis of CRC patient samples collected from our center, confirming the reduced expression of *Cldn7* in tumor tissues (Fig. 2D–F). Moreover, Kaplan–Meier survival analysis revealed that low *Cldn7* expression was significantly associated with poor prognosis in CRC patients (Fig. 2B). Immune infiltration analysis showed a negative correlation between *Cldn7* expression and neutrophil infiltration (Fig. 2C). Multiplex immunofluorescence staining further demonstrated that regions with high *Cldn7* expression exhibited enhanced CD8⁺ T cell infiltration and reduced infiltration of CD66B⁺ polymorphonuclear neutrophils (PMNs) (Fig. 2G–I), suggesting a potential role of *Cldn7* in modulating immune cell distribution and function within the CRC tumor microenvironment. Our previous research has partially confirmed that the expression level of Cldn7 is closely associated with tumor staging and metastasis in CRC, further supporting its potential biological role in CRC [30, 31]. To investigate the functional role of Cldn7 in CRC progression, we established two syngeneic tumor-bearing mouse models. One model employed MC38 cells transduced with either a vector control (Ctrl) or Cldn7 overexpression (Cldn7-oe), and the other used CMT93 cells transduced with either a control shRNA (shNC) or Cldn7 knockdown (shCldn7). In the MC38 model, Cldn7 overexpression significantly inhibited tumor growth, reduced tumor weight, and prolonged mouse survival (Fig. 3A–D). In contrast, Cldn7 knockdown in the CMT93 model promoted tumor growth, increased tumor weight, and shortened survival (Fig. 3K–N). Based on our previous single-cell RNA sequencing and multiplex imaging results, we hypothesized that Cldn7 may influence CRC progression by regulating immune cell function within the tumor microenvironment. Flow cytometry analysis revealed that Cldn7 overexpression markedly reduced the infiltration of CD11b⁺ Ly6G⁺ neutrophils, with no obvious effect on other myeloid cell populations (Fig. 3E, Supplementary Fig. 2A–B), and enhanced CD8⁺ T cell infiltration (Fig. 3F). Further analysis demonstrated that CD8⁺ T cells in the Cldn7-overexpressing group exhibited significantly increased production of effector cytokines IFN-γ and TNF-α, indicating enhanced antitumor immune activity (Fig. 3G–I). Moreover, the expression levels of the inhibitory receptors PD-1 and Tim-3 on CD8⁺ T cells were markedly reduced in the Cldn7-oe group (Fig. 3J), suggesting alleviation of T cell exhaustion. Conversely, in the CMT93 model, Cldn7 knockdown led to increased accumulation of CD11b⁺ Ly6G⁺ neutrophils within the tumor (Fig. 3O, Supplementary Fig. 3A–B) and reduced CD8⁺ T cell infiltration (Fig. 3P, Supplementary Fig. 2C). Additionally, IFN-γ and TNF-α production by CD8⁺ T cells was significantly impaired in the shCldn7 group (Fig. 3Q–S, Supplementary Fig. 3C), indicating weakened effector function. Inhibitory receptor analysis further revealed that PD-1 and Tim-3 expression was elevated in CD8⁺ T cells upon Cldn7 knockdown (Fig. 3T), suggesting enhanced T cell exhaustion. To further elucidate the role of tumor-derived *Cldn7* in regulating PMN infiltration and malignant progression, we performed immune cell depletion experiments in the CMT-93 tumor model (Fig. 4A). In mice with *Cldn7* knockdown, depletion of Ly6G⁺ neutrophils using anti-Ly6G antibodies abolished the pro-tumor effects of *Cldn7* loss, resulting in tumor growth comparable to the *Cldn7*-oe group (Fig. 4B–D). Moreover, when CD8⁺ T cells were depleted using anti-CD8 antibodies, the tumor-promoting effect of *Cldn7* knockdown was markedly attenuated, restoring tumor growth to levels observed in the shNC + anti-CD8 control group (Fig. 4I–K). Flow cytometry showed that Ly6G⁺ cell depletion in *Cldn7*-deficient tumors significantly enhanced CD8⁺ T cell activation, as evidenced by increased IFN-γ (Fig. 4E) and TNF-α (Fig. 4F–H) expression. In summary, loss of *Cldn7* facilitates CRC progression by suppressing CD8⁺ T cell cytotoxicity in a PMN-dependent manner. These findings highlight *Cldn7* as a key modulator of the immune microenvironment in CRC and suggest its potential as a therapeutic target for restoring anti-tumor immunity.

### *Cldn7* deficiency mediates PMN recruitment and phenotypic conversion through the NF-κB/CXCL1 pathway

**Fig. 5:**
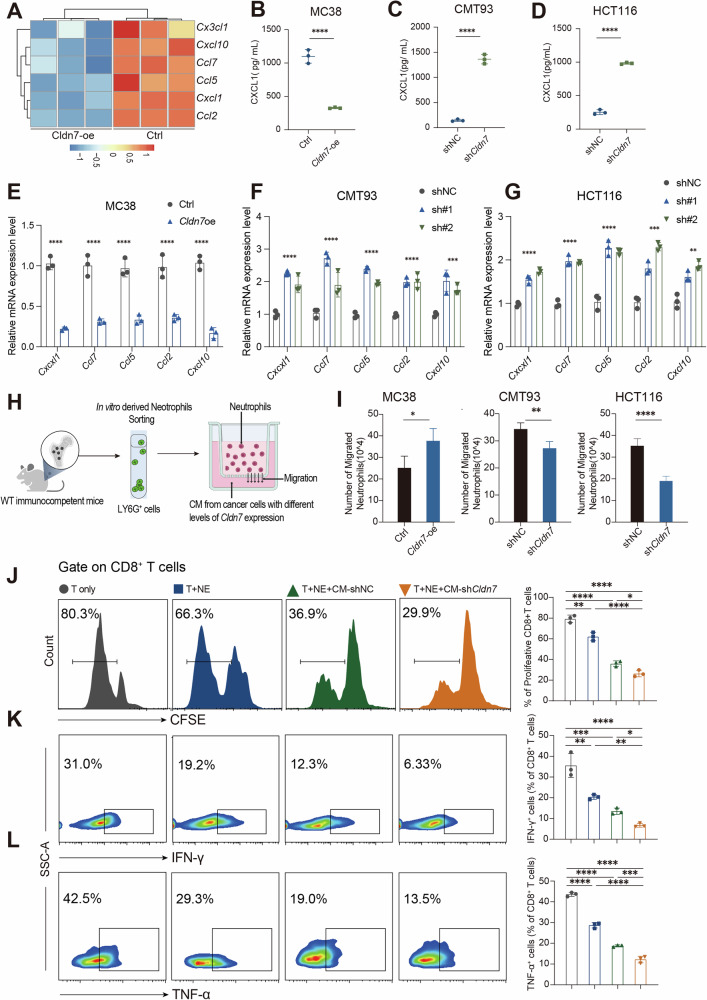
*Cldn7* deficiency recruits PMN aggregation and promotes their phenotypic transformation. **A** Heatmap of RNA-seq data showing that overexpression of *Cldn7* (*Cldn7*-oe) in MC38 cells significantly downregulates the expression of several chemokines involved in neutrophil recruitment, including CX3CL1, CXCL10, CCL7, CXCL1, and CCL2, compared to control (ctrl) cells (*n* = 3 per group). **B**–**D** ELISA analysis of CXCL1 secretion in MC38, CMT93, and HCT116 models. *Cldn7* overexpression markedly reduces CXCL1 levels, while *Cldn7* knockdown (sh*Cldn7*) increases it secretion. **E**–**G** qPCR analysis of chemokine expression in cell lines with different levels of *Cldn7* expression confirms that *Cldn7* overexpression suppresses genes such as CX3CL1, CXCL10, and CCL7. **H** Schematic of the neutrophil migration assay: neutrophils were exposed to conditioned media (CM) from tumor cells with varying *Cldn7* expression to assess their migratory response. **I** Quantification of neutrophil migration in response to CM derived from MC38, CMT93, and HCT116 cells with different *Cldn7* levels. High *Cldn7* expression impairs neutrophil migration, whereas low *Cldn7* expression enhances it. **J** In vitro co-culture assay: CD8^+^ T cells were cultured with neutrophils (T + NE) in the presence of CM from either shNC or sh*Cldn7* tumor cells. CFSE-based proliferation analysis revealed that CM from *Cldn7*-deficient cells suppresses CD8^+^ T cell proliferation. **K**, **L** Neutrophils pre-conditioned with CM from *Cldn7*-deficient cells reduced the proportion of IFN-γ^+^CD8^+^ T cells and TNF-α^+^ CD8 +T cells, suggesting a shift toward an immunosuppressive neutrophil phenotype.

**Fig. 6:**
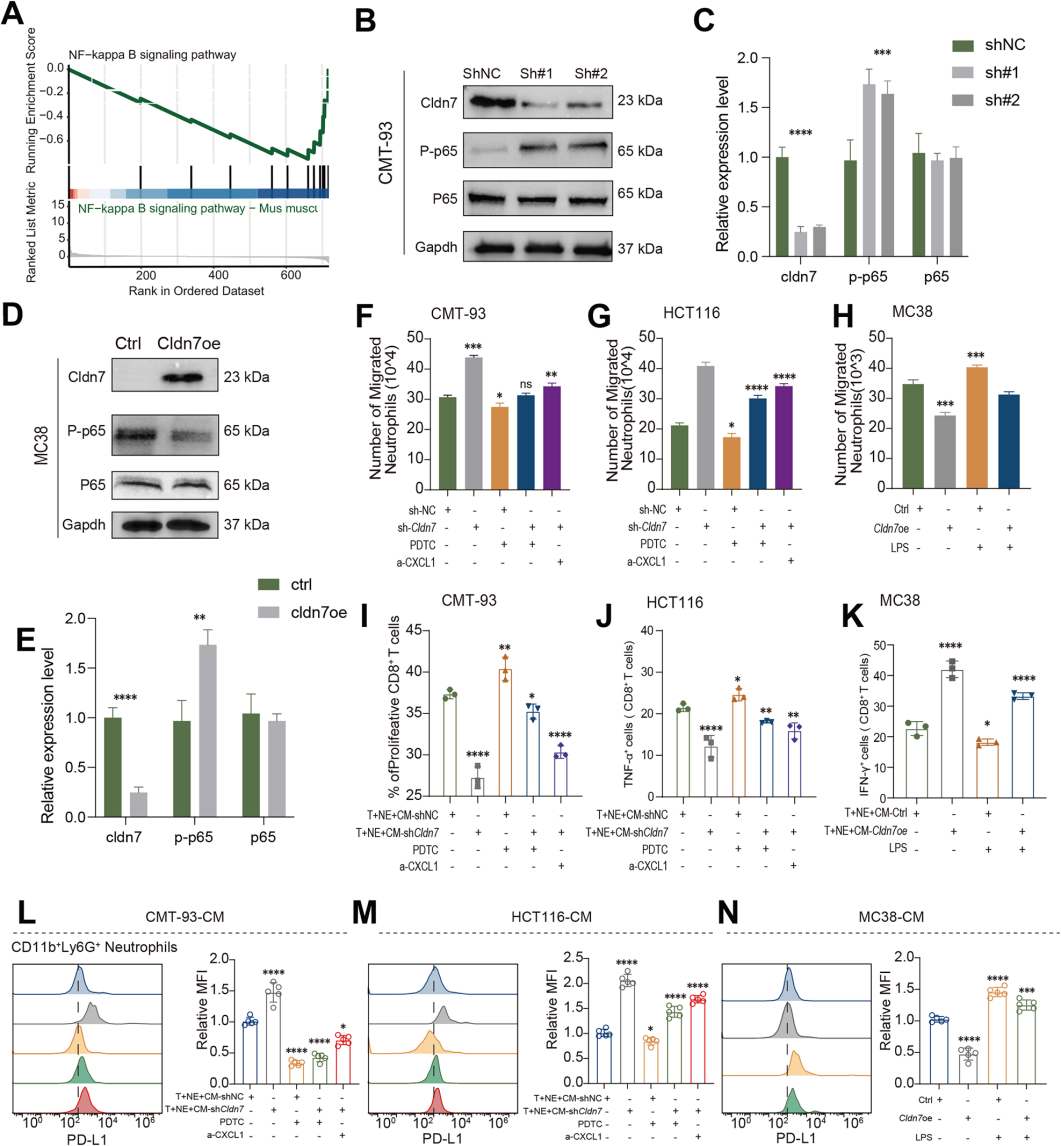
*Cldn7* modulates the NF-kappa B/CXCL1 axis to influence neutrophil recruitment and CD8^+^ T cell activation. **A** Gene Set Enrichment Analysis (GSEA) showed that *Cldn7 overexpression* is associated with suppression of NF-κB signaling pathway activity. Western blot analysis demonstrated a negative correlation between *Cldn7* expression and the levels of key NF-κB signaling proteins, including phospho-p65 (P-p65) and p65, in CMT-93 (**B**, **C**) and MC38 (**D**, **E**) cells. In CMT-93 (**F**), HCT116 (**G**), and MC38 (**H**) cells, CD8+ T cells were co-cultured with neutrophils and treated with either NF-κB pathway activators or inhibitors. The results showed that *Cldn7* deficiency suppressed CD8+ T cell proliferation and activation, as indicated by the proportions of TNF-α⁺ and IFN-γ⁺ CD8+ T cells. These effects were partially reversed by NF-κB activators and enhanced by NF-κB inhibitors. Neutrophil migration assays revealed that *Cldn7* overexpression inhibited neutrophil migration in response to conditioned media from CMT-93 (**I**), HCT116 (**J**), and MC38 (**K**) cells. This inhibitory effect was modulated by the NF-κB pathway: NF-κB activators restored neutrophil migration, while inhibitors further enhanced the suppression. **L**–**N** Analysis of PD-L1 expression in neutrophils cultured with conditioned media from CMT-93, HCT116, and MC38 cells indicated that *Cldn7* knockdown increased of PD-L1 expression in neutrophils.

To further investigate the effect of tumor-derived *Cldn7* on the recruitment of PMN, we performed transcriptome sequencing on the Ctrl/*Cldn7*-oe mc38 cell lines. The results showed that *Cldn7* overexpression significantly downregulated the expression of several chemokines related to PMN recruitment, including *Cxcl1*, *Ccl2*, *Cxcl10* and *Ccl7* (Fig. 5A). Further ELISA validation demonstrated that CXCL1 secretion was significantly reduced in MC38 Cldn7-overexpressing cells, while it was significantly increased upon Cldn7 knockdown (Fig. 5B–D). Additionally, qPCR results confirmed that Cldn7 negatively regulates the expression of these chemokines in various CRC cell lines MC38, CMT93, and HCT116 (Fig. 5E–G). The in vitro neutrophil chemotaxis assay showed that conditioned medium (CM) from Cldn7-low expression cells significantly enhanced PMN migration (Fig. 5H, I), with no obvious impact on PMN apoptosis (Supplementary Fig. 4A). This suggests that the increased PMN infiltration in CRC primarily results from enhanced chemotaxis due to the downregulation of tumor-derived *Cldn7*. Functional experiments further demonstrated that PMNs induced by *Cldn7* deficiency significantly inhibited CD8⁺ T cell proliferation and the secretion of IFN-γ and TNF-α (Fig. 5J–L). These results indicate that *Cldn7*-deficient CRC cells promote PMN recruitment and induce their conversion to a pro-tumor phenotype, thereby creating an immunosuppressive microenvironment that enhances CRC progression by inducing CD8⁺ T cell exhaustion. Next, we performed further analysis of transcriptome data from MC38 *Cldn7*-oe/sh cell lines. Gene Set Enrichment Analysis (GSEA) revealed that the NF-κB signaling pathway was significantly suppressed in *Cldn7*-overexpressing cells, suggesting that *Cldn7* may exert its effects by inhibiting NF-κB pathway activation (Fig. 6A, Supplementary Fig. 5A, B). Further Western blot experiments showed that NF-κB activity was elevated in *Cldn7* knockdown CMT93 cells (Fig. 6B, C), while overexpression of *Cldn7* inhibited this effect (Fig. 6D, E). Migration assays showed that CM from *Cldn7* knockdown CMT93 and HCT116 cells significantly enhanced PMN migration, an effect that could be partially reversed by the NF-κB inhibitor PDTC or CXCL1-neutralizing antibodies. Conversely, CM from *Cldn7*-oe MC38 cells significantly suppressed PMN migration, even under LPS-induced NF-κB activation conditions (Fig. 6F–H). These results suggest that *Cldn7* negatively regulates PMN recruitment by inhibiting NF-κB-driven chemokine secretion (e.g., CXCL1). Further in vitro co-culture experiments showed that PMNs treated with CM from *Cldn7* knockdown cells significantly inhibited CD8⁺ T cell proliferation (Fig. 6I) as well as the secretion of cytokines such as IFN-γ (Fig. 6J) and TNF-α (Fig. 6K). The immunosuppressive effect on CD8⁺ T cells could be partially reversed by PDTC or CXCL1-neutralizing antibodies, further confirming the key role of the NF-κB/CXCL1 axis in this process. Additionally, *Cldn7* knockdown also induced an increase in PD-L1 expression on PMN surfaces, an effect that could be reversed by PDTC or CXCL1-neutralizing antibodies (Fig. 6L, M). Even under LPS-induced NF-κB activation conditions, *Cldn7*-oe still suppressed PD-L1 expression (Fig. 6N), further suggesting that *Cldn7* modulates PMN’s immunosuppressive phenotype through negative regulation of the NF-κB/CXCL1 axis.

### Tumor-derived *Cldn7* regulates metabolic reprogramming of neutrophils in the CRC microenvironment

**Fig. 7:**
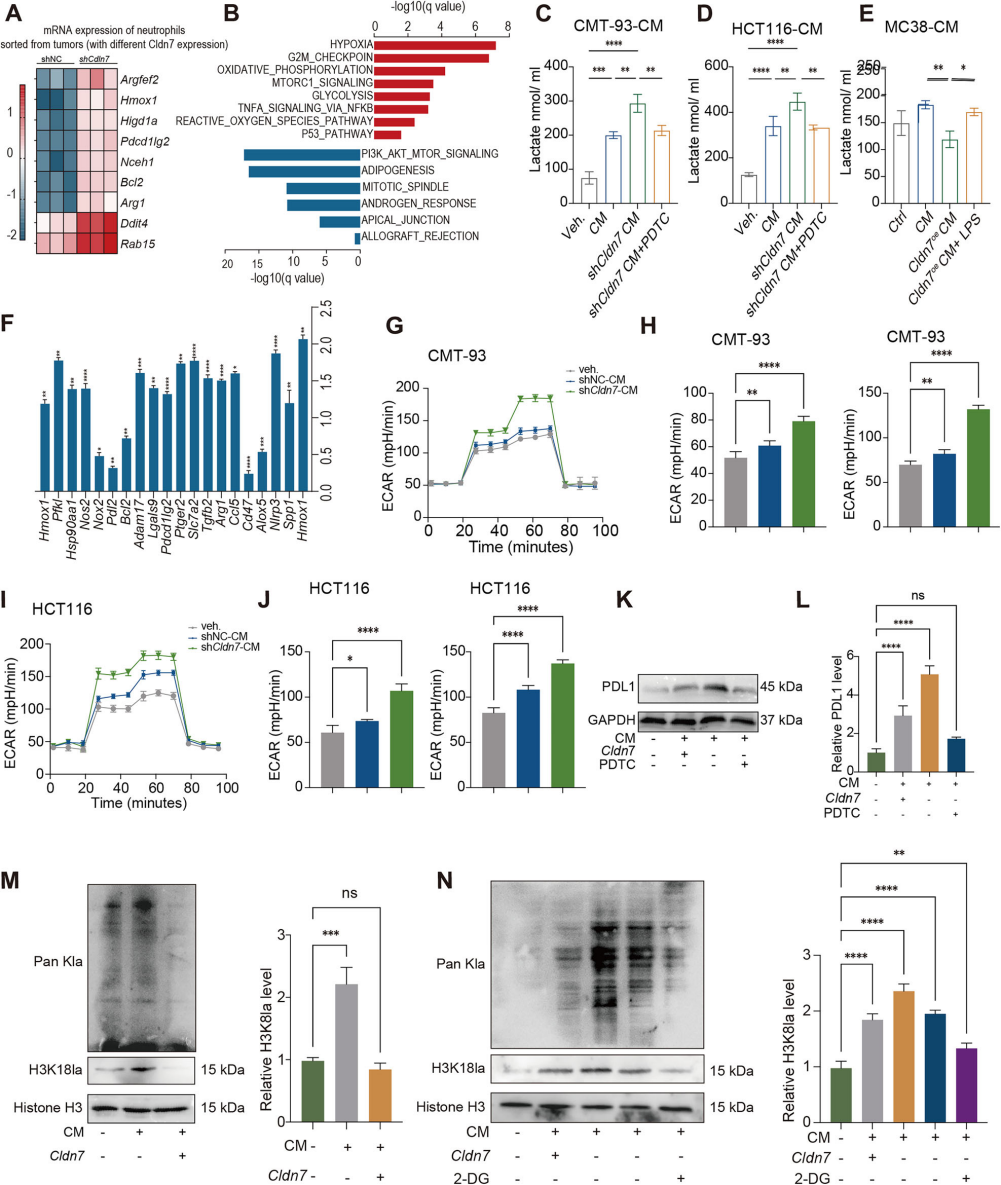
Tumor-derived *Cldn7* modulates metabolic reprogramming and immune-suppressive phenotype of neutrophils in the CRC microenvironment. **A** RNA expression analysis of neutrophils (CD11b^+^ Ly6G^+^) isolated from the tumor microenvironment of *Cldn7*-overexpressing and *Cldn7*-shRNA tumors revealed differential expression of genes associated with metabolic reprogramming and immune suppression. **B** Gene ontology (GO) enrichment analysis indicated significant enrichment of pathways such as oxidative phosphorylation and TNF signaling in neutrophils from *Cldn7*-shRNA tumors. Lactate production in neutrophils co-cultured with conditioned media (CM) from CMT-93 (**C**), HCT116 (**D**), and MC38 (**E**) tumor cell lines was significantly increased in the *Cldn7*-shRNA groups, which was reversed by treatment with the NF-κB pathway inhibitor PDTC (**C**, **D**). **F** qPCR analysis of neutrophils after co-culture with tumor CM showed that glycolysis-related and anti-apoptotic genes were markedly upregulated under *Cldn7*-shRNA conditions. **G**–**J** ECAR (extracellular acidification rate) measurements of neutrophils cultured in CM from CMT-93 (**G**), HCT116 (**I**), and HCT116-*Cldn7*-shRNA (**J**) demonstrated enhanced glycolytic activity in the *Cldn7*-shRNA groups. **K** Western blot analysis showed that PD-L1 protein expression was elevated in neutrophils exposed to CM from *Cldn7*-shRNA tumor cells. **L** Quantification of PD-L1 expression confirmed significant upregulation in neutrophils treated with CM from *Cldn7*-shRNA tumors. **M**, **N** Tumor-derived *Cldn7* regulates histone lactylation to mediate neutrophil metabolic reprogramming. Western blot analysis showed increased levels of histone lactylation markers, including Pan-Kla and H3K18la, in neutrophils co-cultured with CM from *Cldn7*-overexpressing MC38 cells (**M**). Conversely, CM from *Cldn7* knockdown CMT-93 cells also led to elevated Pan-Kla and H3K18la expression in neutrophils, which was reversed by treatment with the glycolysis inhibitor 2-DG (**N**). These results suggest that tumor-derived *Cldn7* alters neutrophil metabolic status through histone lactylation, thereby promoting their immune-suppressive phenotype.

To investigate how tumor-derived *Cldn7* expression modulates neutrophil function and contributes to immune suppression in CRC, we performed bulk RNA sequencing on CD11b^+^Ly6G^+^ neutrophils sorted from tumor tissues of CMT93 tumor-bearing mice with either shNC or sh*Cldn7*. The results revealed that in neutrophils from the sh*Cldn7* group, genes associated with metabolic reprogramming and immunosuppression were significantly upregulated, including anti-apoptotic genes such as *Argef2, Hmox1*, *Higd1a*, *Arg1*, and *Bcl2* (Fig. 7A). Gene Set Enrichment Analysis (GSEA) further indicated enhanced metabolic activity in tumor-derived neutrophils from the sh*Cldn7* group, involving key pathways such as hypoxia, oxidative stress, glycolysis, and the reactive oxygen species (ROS) pathway (Fig. 7B).To validate the effect of *Cldn7* on neutrophil metabolism, we established *Cldn7* knockdown and overexpression models in CMT-93, HCT116, and MC38 cell lines, and collected their conditioned media (CM) for co-culture with in vitro–sorted neutrophils. CM from *Cldn7* knockdown cells significantly increased lactate production in neutrophils, an effect that could be reversed by the NF-κB pathway inhibitor (PDTC). Conversely, CM from *Cldn7*-overexpressing cells suppressed lactate production, and this suppression was counteracted by the NF-κB activator (LPS) (Fig. 7C–E). qPCR analysis revealed that neutrophils cultured in *Cldn7* knockdown tumor CM exhibited marked upregulation of genes associated with glycolysis, anti-apoptosis, and immunosuppression (Fig. 7F). Moreover, extracellular acidification rate (ECAR) assays confirmed that CM from *Cldn7* knockdown tumors promoted increased glycolytic activity in neutrophils (Fig. 7G–J). In parallel, Western blot analysis showed significantly elevated PD-L1 expression in neutrophils under *Cldn7* knockdown conditions, indicating a more immunosuppressive phenotype (Fig. 7K, L). To further explore whether metabolic reprogramming regulates neutrophil immune function via epigenetic mechanisms, we assessed histone lactylation levels. Neutrophils under Cldn7 knockdown conditions exhibited increased levels of Pan-Kla and H3K18la, both of which were markedly reduced by treatment with the glycolysis inhibitor 2-DG. In contrast, CM from *Cldn7*-overexpressing cells suppressed histone lactylation in neutrophils (Fig. 7M, N). In summary, these results demonstrate that tumor-derived *Cldn7* modulates neutrophil glycolytic metabolism via the NF-κB signaling pathway and further regulates their immunosuppressive phenotype through histone lactylation. This mechanism highlights a novel role for *Cldn7* in orchestrating immune modulation within the CRC tumor microenvironment.

### Overexpression of *Cldn7* enhances the efficacy of anti-PD-1 therapy in a subcutaneous tumor model of CRC cells

**Fig. 8:**
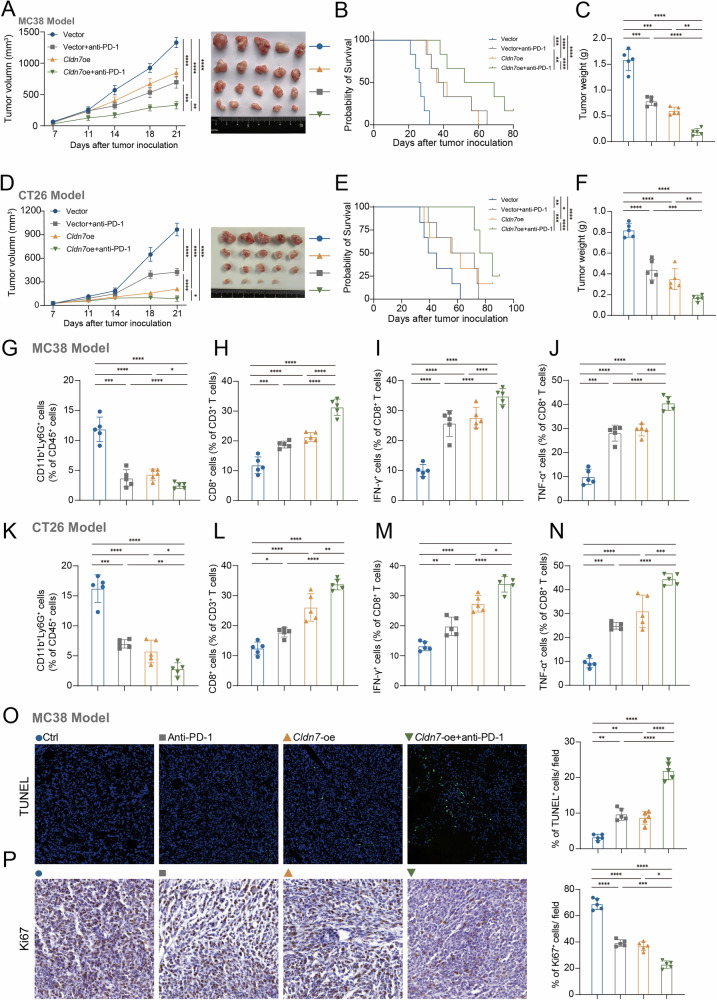
*Cldn7* overexpression enhances the therapeutic effect of PD-1 antibody in MC38 and CT26 tumor models. Tumor volume growth curves of MC38 (**A**) and CT26 (**D**) tumor-bearing mice under different treatment conditions, including Vector, Vector + anti-PD-1, *Cldn7* overexpression (*Cldn7*-oe), and *Cldn7*-oe + anti-PD-1 groups. Tumor volumes were measured on days 7, 11, 14, 18, and 21. *Cldn7*-oe significantly suppressed tumor growth, and this effect was further enhanced by anti-PD-1 treatment. Survival curves of MC38 (**B**) and CT26 (**E**) tumor-bearing mice across the different treatment groups. The *Cldn7*-oe + anti-PD-1 group exhibited significantly prolonged survival compared to controls. Final tumor weights of MC38 (**C**) and CT26 (**F**) models at the end of the experiment. Combined *Cldn7* overexpression and PD-1 antibody treatment led to a marked reduction in tumor burden. Flow cytometry analysis of the proportion of CD11b⁺Ly6G⁺ myeloid-derived suppressor cells (MDSCs) within CD45⁺ cells in MC38 (**G**) and CT26 (**K**) tumor tissues. MDSC infiltration was significantly reduced in the *Cldn7*-oe + anti-PD-1 group. Flow cytometric analysis of CD8⁺ T cells within CD3⁺ T cells in MC38 (**H**) and CT26 (**L**) tumor tissues. *Cldn7* overexpression significantly increased CD8⁺ T cell infiltration, particularly in the combination therapy group. Proportion of IFN-γ⁺ cells within CD8⁺ T cells in MC38 (**I**) and CT26 (**M**) models, showing that *Cldn7* overexpression combined with anti-PD-1 treatment significantly enhanced CD8⁺ T cell activation. Proportion of TNF-α⁺ cells within CD8⁺ T cells in MC38 (**J**) and CT26 (**N**) tumors. TNF-α expression was notably upregulated in the *Cldn7*-oe + anti-PD-1 group, indicating enhanced CD8⁺ T cell effector function. TUNEL (**O**) and Ki67 (**P**) immunohistochemical staining of MC38 tumor tissues to assess tumor cell apoptosis and proliferation. Results showed increased apoptosis and decreased proliferation in the *Cldn7*-oe + anti-PD-1 group. Statistical significance: *p < 0.05, **p < 0.01, ***p < 0.001, ****p < 0.0001.

Based on in vivo and in vitro evidence showing that *Cldn7* deficiency induces immune evasion by promoting PMN aggregation and their conversion to a pro-tumor phenotype, we aimed to investigate whether increasing *Cldn7* expression could enhance the response to immunotherapy. In this experiment, MC38 and CT26 cells were injected subcutaneously into immunocompetent mice, which were then divided into four groups: control (Ctrl), anti-PD-1 treatment alone, *Cldn7*-oe, and combination treatment with *Cldn7* overexpression and anti-PD-1 (*Cldn7*-oe + anti-PD-1). Compared to the other groups, tumor growth was significantly reduced in the *Cldn7*-oe + anti-PD-1 group (Fig. 8A, D). Survival analysis revealed a significant prolongation of survival time in the combination treatment group (Fig. 8B, E), accompanied by a marked reduction in tumor weight (Fig. 8C, F). Flow cytometry analysis showed that Cldn7 overexpression combined with anti–PD-1 therapy significantly reduced the infiltration of CD11b⁺Ly6G⁺ neutrophils (Fig. 8G, K and Supplementary Fig. 7A, D), while notably increasing CD8⁺ T cell infiltration, particularly the proportion of IFN-γ⁺ and TNF-α⁺ CD8⁺ T cells (Fig. 8H–J, L–N). Moreover, this combination treatment significantly enhanced the cytotoxic functionality of CD8⁺ T cells, as evidenced by elevated levels of effector cytokine production. In parallel, the expression of exhaustion markers such as PD-1 and TIM-3 on CD8⁺ T cells was markedly reduced, indicating a substantial improvement in T cell functional status and reinforcing their anti-tumor efficacy (Supplementary Fig. 7B, C, E, F). TUNEL staining and Ki67 IHC staining indicated increased apoptosis and reduced proliferation in the tumors of the combination treatment group (Fig. 8O, P).

**Fig. 9:**
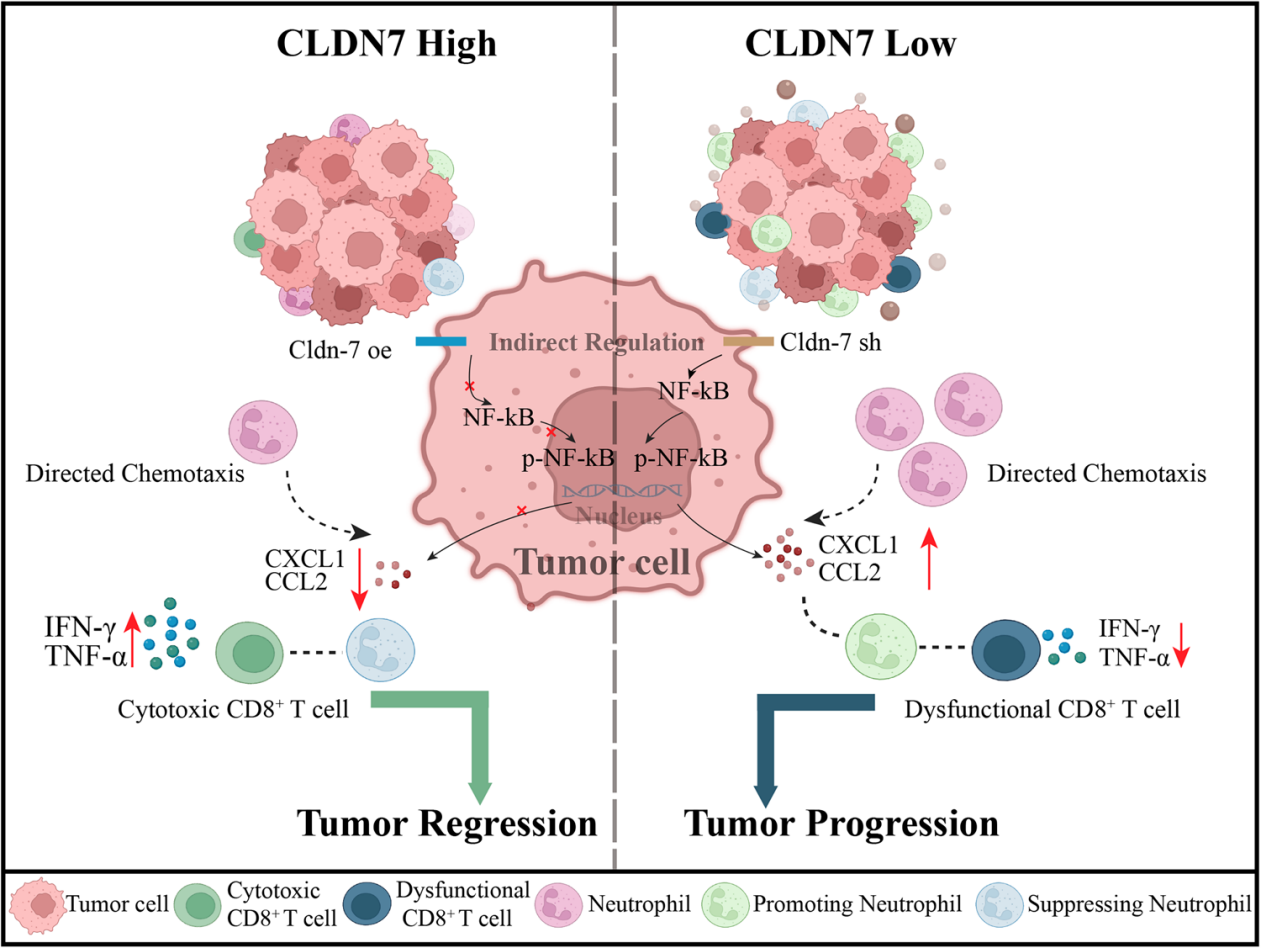
Model of colorectal cancer showing that under conditions of *Cldn7* deficiency, recruited neutrophils convert to a pro-tumor phenotype, further inhibiting CD8^+^ T cell function. Left (*Cldn7* high): With high *Cldn7* expression (*Cldn7* overexpression, *Cldn7*-oe), the NF-κB signaling pathway is suppressed, leading to reduced secretion of neutrophil-attracting chemokines such as CXCL1 and CCL12, thereby limiting neutrophil recruitment to the tumor site. The reduction in neutrophils prevents suppression of CD8^+^ T cells and also limits the conversion of neutrophils to a pro-tumor phenotype. This allows CD8^+^ T cells to exert their cytotoxic functions effectively, producing IFN-γ and TNF-α, which enhances the anti-tumor immune response and leads to colorectal cancer regression. Right (*Cldn7* low): Under conditions of *Cldn7* deficiency (*Cldn7* knockdown, *Cldn7*-sh), the NF-κB signaling pathway is activated, leading to increased secretion of CXCL1 and CCL12, which promotes neutrophil recruitment to the tumor site. These recruited neutrophils convert to a pro-tumor phenotype and suppress CD8^+^ T cell activation and function (e.g., reduced production of IFN-γ and TNF-α), thereby creating an immunosuppressive microenvironment that promotes colorectal cancer progression. This model suggests that high *CLDN7* expression indirectly supports CD8^+^ T cell anti-tumor function by inhibiting neutrophil recruitment and their conversion to a pro-tumor phenotype. In contrast, *Cldn7* deficiency promotes pro-tumor neutrophil recruitment and suppresses CD8^+^ T cell function, creating an immunosuppressive microenvironment conducive to colorectal cancer growth. This figure was created using BioRender.com.

In conclusion, these findings demonstrate that *Cldn7* overexpression enhances the efficacy of anti-PD-1 therapy in CRC by promoting anti-tumor immunity. In both MC38 and CT26 models, *Cldn7* overexpression combined with anti-PD-1 treatment significantly inhibits tumor growth, reduces tumor weight, and prolongs survival. This combined treatment decreases the infiltration of immunosuppressive CD11b^+^ Ly6G^+^ neutrophils while increasing CD8^+^ T cell infiltration and activation, as evidenced by higher IFN-γ and TNF-α production. These results suggest that targeting *Cldn7* may help create a more immune-supportive microenvironment, thereby enhancing the effectiveness of immune checkpoint therapy in CRC (Fig. 9)

## Discussion

Colorectal cancer (CRC) is a common malignant tumor, ranking third in incidence (6.1%) and second in mortality (9.4%) among all cancers [32]. Due to the diversity and heterogeneity of CRC, effective treatment strategies have been relatively limited in recent years, aside from surgical resection. With the revolutionary advancements brought by immunotherapy in postoperative treatment, combined with radiotherapy, chemotherapy, and targeted therapy, CRC treatment has entered a new phase [33]. Increasing evidence suggests that tumor-associated TANs play a significant role in modulating antitumor responses [20, 34, 35]. Due to their high functional heterogeneity, pro-tumor TANs are considered a critical factor limiting the efficacy of cancer immunotherapy [36–38]. However, the lack of markers or targets to specifically identify pro-tumor TANs poses a challenge in developing strategies to selectively eliminate these cell [21, 39–41].

Neutrophils undergo profound metabolic adaptations within the tumor, ultimately leading to distinct pro-tumor or anti-tumor phenotypes [42–44]. This study thoroughly investigates how tumor-derived *Cldn7* alters the CRC tumor microenvironment, inducing functional and metabolic reprogramming of polymorphonuclear neutrophils (PMN), which in turn affects CD8^+^T cell function and influences the malignant progression of CRC. Our previous research, utilizing the *Cldn7*^fl/fl;Villin-CreERT2^ mouse model, revealed that the loss of *Cldn7* is associated with exacerbated colonic inflammation and carcinogenesis, closely linked to increased neutrophil infiltration and elevated chemokine expression [10, 28, 31]. Furthermore, single-cell data analysis of clinical surgical specimens and transcriptome sequencing of tumor cells indicated that changes in the expression level of tumor-derived *Cldn7* can significantly regulate the secretion of chemokines related to neutrophil recruitment by tumor cells. In vitro experiments confirmed that tumor cells with knocked-down *Cldn7* expression promote PMN migration by enhancing the secretion of related chemokines. Conversely, *Cldn7* overexpression significantly reduces this chemotactic ability. These findings suggest that *Cldn7* modulates the directional migration of PMN by regulating the expression or activity of chemokines, thereby influencing the dynamics of immune cells within the tumor microenvironment.

Moreover, this study found that changes in *Cldn7* expression not only affect PMN function but also contribute to reshaping their metabolic phenotype. PMN within the CRC microenvironment with low *Cldn7* expression exhibit enhanced glycolytic activity, upregulation of anti-apoptotic and immunosuppressive genes, and a shift toward a glycolysis-driven functional state through histone lactylation, ultimately displaying an immunosuppressive neutrophil phenotype. Additionally, neutrophils in the *Cldn7*-deficient CRC microenvironment suppress CD8⁺ T cell function by reducing their proliferative capacity and antitumor activity—effects that can be partially reversed by inhibiting the NF-κB signaling pathway.

Although PD-1/PD-L1 blockade therapy has been widely used in clinical settings, the fundamental mechanisms underlying the restoration of dysfunctional CD8^+^ T cells in cancer remain incompletely understood [45, 46]. Increasing evidence suggests that the heterogeneity of dysfunctional CD8^+^ T cells in cancer is a major obstacle to the success of PD-1/PD-L1 blockade therapy [47, 48]. In our study, after anti-PD-1 treatment using the MC38 and CT26 mouse subcutaneous tumor models, we observed that mice with overexpressed *Cldn7* exhibited better therapeutic outcomes following anti-PD-1 treatment, with slower tumor growth rates and significantly prolonged survival. These findings indicate that *Cldn7* enhances the response to PD-1 inhibitors by improving the tumor microenvironment, specifically, it reduces the infiltration of immunosuppressive TAN and enhances the function of tumor-infiltrating CD8^+^ T cells, thereby improving the response to PD-1 inhibitors (Fig. 9).

Neutrophils are part of the bone marrow and stromal cell network, express PDL1, and drive immune checkpoint engagement and T cell exhaustion [44]. In this study, we investigated whether tumor-derived *Cldn7* deletion recruits PMN infiltration, promoting their transition to a pro-tumor phenotype and thereby forming an immunosuppressive microenvironment. Through single-cell sequencing, clinical sample validation, and in vivo and in vitro experiments, we found that *Cldn7* deletion leads to increased recruitment of CD11b^+^ Ly6G^+^ neutrophils in the CRC microenvironment and accelerates CRC malignant progression. *Cldn7* deletion indirectly activates the NF-κB signaling pathway, resulting in increased secretion of PMN recruitment-related chemokines such as CXCL1. Inhibiting the NF-κB /CXCL1 axis in *Cldn7* deficiency tumor cells can reduce PMN recruitment, decrease PD-L1 expression on PMN, and reverse the exhausted phenotype of CD8 + T cells, thereby improving the immunosuppressive microenvironment. Additionally, *Cldn7* overexpression can enhance the effectiveness of ICB (immune checkpoint blockade) therapy. Overall, our experimental results suggest that *Cldn7* deletion plays an initiating role and has immunoregulatory functions in CRC malignant progression, and targeting related biological processes may enhance CRC immunotherapy efficacy in the future.

## Conclusion

Our experimental results highlight the pivotal role of *Cldn7* in CRC progression by modulating the behavior of immune cells, particularly TAN and CD8^+^ T cells, within the tumor microenvironment, thereby influencing tumor immune evasion and antitumor immune responses. This provides a deeper understanding of how tumors promote their survival and progression by regulating local immune cells. The immunosuppressive TAN formed under the condition of *Cldn7* deficiency will become the focus and challenge of subsequent research.

## Material and methods

### Ethics approval and consent to participate

All methods in this study were performed in accordance with the relevant guidelines and regulations. The study was approved by the Ethics Committee of Beijing Shijitan Hospital Institutional Review Board (approval number: sjtkyll-1x-2021(105)). Tumor specimens from colorectal cancer radical resection were collected at the Department of Gastrointestinal Oncology, Beijing Shijitan Hospital, Capital Medical University, for single-cell sequencing or embedding in 4% paraformaldehyde. After obtaining informed consent, whole blood samples were collected from healthy volunteers for neutrophil separation. All animal experiments were conducted in compliance with the ARRIVE guidelines and approved by the Animal Ethics Committee of Beijing Shijitan Hospital Institutional Review Board (approval number: sjtkyll-1x-2021(105)). Informed consent was obtained from all participants involved in the study. For any identifiable human images included in this article, written informed consent for publication of the images was obtained from the participants, which is separate from the consent to participate.

### Cell cultures

MC38, CMT-93, CT26, and HCT116 cells were obtained from the American Type Culture Collection (ATCC, USA). All cell lines were maintained in high-glucose DMEM (GMEM, GIBCO, USA) supplemented with 10% fetal bovine serum (GIBCO) and penicillin/streptomycin, at 37 °C with 5% CO_2_. Cells were routinely tested for mycoplasma contamination. For immune cell cultures, primary cells were isolated from peripheral sources (such as blood or bone marrow) or spleen, and immediately used for functional analyses, such as neutrophil-T cell co-cultures or neutrophil migration, with detailed methods found in the supplementary materials.

### Statistical analysis

Flow cytometry data were analyzed using FlowJo software (V.12; Tree Star), and graphs and statistical analyses were generated using GraphPad Prism (V.9). Single-cell and bulk transcriptome data analysis were conducted using R 4.2.0. One-way or two-way analysis of variance (ANOVA) was used for comparisons among more than two groups, while an unpaired two-tailed *t*-test was used for comparisons between only two groups. A *p*-value of <0.05 was considered statistically significant (*p < 0.05, **p < 0.01, ***p < 0.001). Mouse survival was assessed using the Kaplan–Meier method and analyzed using the Mantel–Cox log-rank test. All experiments were performed at least three times, with *n* representing biological replicates.

## Supplementary information

Supplementary materials

Original data for western blot

Original data for qpcr

## Data Availability

The TCGA data used in this study were downloaded from The Cancer Genome Atlas (TCGA) database (https://portal.gdc.cancer.gov/). All other datasets generated and/or analyzed during the current study are available from the corresponding author upon reasonable request.
