# Supplementary material for: Claudin-7 deficiency induces metabolic reprogramming of neutrophils in the colorectal cancer microenvironment
Source: Cell Death Dis. 2025 Oct 16;16(1):728. doi: 10.1038/s41419-025-08064-3 (PMC12533015; doi:10.1038/s41419-025-08064-3)
Supplement: Supplementary file 1 — Supplementary materials [file 41419_2025_8064_MOESM1_ESM.pdf]

Supplementary materials

Supplementary methods

1. Single cell analysis

Surgical resection tissues from five cases of colorectal cancer without preoperative treatment were immediately placed on ice at 4°C after excision. Fresh tissues were enzymatically dissociated into single-cell suspensions and submitted to Shanghai Liebing Biotechnology Co., Ltd. (Beijing, China) for single-cell RNA sequencing (scRNA-seq). After cell counting, libraries were constructed using the Chromium platform from 10X Genomics, and sample demultiplexing, barcode processing, sequence alignment, and unique molecular identifier (UMI) counting were performed using the Cell Ranger pipeline (v1.2). The raw gene expression matrix was processed in R (v4.2.0) using the Seurat package (v4.2.0)(1). The quality control workflow included: removal of doublets using the DoubletFinder package(2); elimination of ambient RNA contamination using the DecontX package(3); and retention of high-quality cells expressing 300–7000 genes, with mitochondrial gene content <20% and hemoglobin gene content <10%. The top 2000 highly variable genes were identified using the FindVariableFeatures function, followed by normalization with the ScaleData function. Batch effects were corrected using the harmony package(4). Principal component analysis (PCA) was performed based on the highly variable genes, and the top 30 principal components were used for dimensionality reduction via the Uniform Manifold Approximation and Projection (UMAP) algorithm. Differentially expressed genes for each cell subtype were identified using the FindAllMarkers function. Cells type annotation was based primarily on the expression patterns of canonical markers for major cell types, as well as known or newly identified markers of subpopulations. Published cell annotations served as the main reference for defining major cell types(5-8). Copy number variations (CNVs) in epithelial cells were analyzed using the *InferCNV* tool to distinguish malignant from non-malignant subpopulations (https://github.com/broadinstitute/inferCNV). Additionally, intercellular communication networks were inferred using the *'CellChat'* package(9).

2. Extracellular Acidification Rate

The ECAR (Extracellular Acidification Rate) assay is used to assess the glycolytic capacity of cells and is conducted using the Seahorse XF Analyzer platform. Initially, conditional media (CM) are collected from tumor cells with varying levels of cldn7 expression. Tumor cells are cultured until 70–80% confluency, after which the medium is replaced with serum-free RPMI or DMEM. The conditioned media are collected after 24–48 hours by centrifugation at 3000 rpm for 30 minutes, followed by filtration through a 0.22 μm membrane to remove cellular debris. Neutrophils (Neu) are then enriched from mouse bone marrow using Ly6G magnetic beads (BioLegend) and seeded at 4×10^4 cells/well in a 96-well Seahorse plate. These

neutrophils are cultured in the collected CM from different tumor sources for 6 hours to allow metabolic conditioning. After this incubation, the CM-conditioned neutrophils are transferred to a specialized Seahorse microplate, and the sensor probes are hydrated in advance. Subsequently, glucose, oligomycin, and 2-deoxyglucose (2-DG) are sequentially injected to initiate glycolysis, inhibit mitochondrial ATP production, and confirm the glycolytic source of acidification, respectively. The assay is performed in a CO_2-free environment using Seahorse XF base medium without bicarbonate. ECAR values are measured at each stage to calculate key glycolytic parameters, including glycolysis, glycolytic capacity, glycolytic reserve, and non-glycolytic acidification, providing a comprehensive evaluation of cellular metabolic activity.

3. Lactate test

After enriching neutrophils from mouse bone marrow using the aforementioned method, the cells were co-cultured with conditional media (CM). Following incubation, the neutrophils were harvested, and lactate levels were measured using the lactate assay kit (catalog number P0392S) from Beyotime Biotechnology. All experimental procedures were performed in strict accordance with the manufacturer's instructions.

4. Generation of Stable Cell Lines via Lentiviral Infection

Experimental procedures were performed according to previously published literature25. The sequences of the vectors used are as follows:

Serial Number	Target Sequence
Cldn7-Mus-76737	GCGGGCGACAACATCATCACA
Cldn7-Mus-76513	GCAGGCCACTCGAGCCTTAAT
Cldn7-Mus-76515	GGAGATGACAAAGCGAAGAAG

5. Mice and in vivo tumor studies

Female C57BL/6j mice aged 6 to 8 weeks were purchased from SPF (Beijing) biotechnology Co. Ltd. (China). All animal experiments were approved by the Ethics Committee of Beijing Shijitan Hospital Institutional Review Board and conducted in accordance with the guidelines for the care and use of laboratory animals. Mice were kept in an SPF environment. For the MC38 and CMT-93 models, C57BL/6J mice were respectively inoculated in situ with 5×105 tumor cells. Tumor volume was measured 2-3 times per week using a digital caliper, and calculated using the formula volume = ($L \times W^2/2$).

6. Elisa

After preparing the cell culture supernatant, collect the supernatant and allow the ELISA kit to equilibrate to

room temperature, dilute the standards as per the ratio, and set up the wells; add 100 μL of standards or samples to each well, incubate at 37°C for 90 minutes, then wash the plates twice; add 100 μL of biotin-antibody working solution to each well, incubate at 37°C for 60 minutes, then wash the plates three times; add 100 μL of SABC working solution to each well, incubate at 37°C for 30 minutes, then wash the plates five times; add 90 μL of TMB substrate to each well, incubate at 37°C in the dark for 10-20 minutes, then add 50 μL of stop solution, and finally read the OD value at 450nm using a microplate reader.

7. Flow cytometry

During the preparation of single-cell suspensions from tumor tissues, tumor tissues from mice at the observation endpoint were dissected, weighed, and photographed. Peripheral tumor tissues were finely minced and added to a digestion solution (serum-free 1640 medium with collagenase V, hyaluronidase, and DNase). The tissues were then digested at 37°C for 2 hours, with pipette mixing every 20 minutes to enhance digestion efficiency. After digestion, the suspension was filtered through mesh and cell strainers to obtain single-cell suspensions, followed by PBS resuspension, red blood cell lysis, and several rounds of centrifugation and washing. Cells were then allocated according to the experimental protocol: part of the sample was used for lymphocyte staining with direct antibody labeling, while another part was used for T cell stimulation, in which FBS and Cell Activation Cocktail were added to the culture medium. After stimulation at 37°C for 5 hours, cells were labeled and stained with antibodies. Finally, the samples were analyzed using a Beckman flow cytometer, and data were processed with FlowJo software. The flow cytometry gating strategy is shown in Supplementary Figure 1, and the list of flow cytometry antibodies used is provided in Supplementary Table 2.

8. Flow cytometry analysis of apoptosis

The cells to be tested are centrifuged at 1000rpm for 5 minutes, the supernatant is discarded, and the cells are washed once with PBS. Add 5 μL of Annexin V and 5 μL of 7AAD antibody, incubate in the dark for 15 minutes, and then proceed with the detection using the flow cytometer.

9. In vitro T cell proliferation and function assay

Mouse T cells were isolated from the spleens of 6-8 weeks-old C57/BL6 mice, and CD8+ T cells were separated using immunomagnetic beads according to the manufacturer's instructions (Biolegend, Cat# 480035). The sorted CD8+ T cells were labeled with CFSE, then seeded into 24-well plates pre-coated with CD3 and CD28, and 2 μg/mL IL-2 was added to maintain the culture. PMNs sorted by immunomagnetic beads were added to conditioned media (CM) from tumor cells with different Cldn7 levels and cultured for 12 hours. These PMNs were then added to the 24-well plates at a 1:1 ratio with CD8+ T cells. After 72 hours of co-culture, Cell Activation Cocktail (with Brefeldin A, Biolegend, Cat# 423304) was added for 5 hours before flow cytometry analysis with specific antibodies.

10. Western blot

The experimental steps for Western blot can be obtained from previously published literature. The immunoreactive bands were imaged using the LI-COR Odyssey Imaging System (LI-COR). The antibodies used in Western blotting are listed in Supplementary Table 3.

11. Multiplex immunofluorescence staining

In the multiplex immunofluorescence staining procedure for tissue sections, paraffin slides were first deparaffinized and rehydrated, followed by antigen retrieval in an EDTA buffer at pH 8.0 using a microwave. After blocking endogenous peroxidase with hydrogen peroxide and sealing with serum or BSA, the slides were incubated overnight with the primary antibody at 4° C. The corresponding HRP-labeled secondary antibody was applied, followed by TSA fluorophore staining steps (e.g., CY3, FITC, CY5) with microwave treatment between rounds to remove previously bound antibodies. DAPI was used for nuclear counterstaining, and autofluorescence was quenched. Finally, slides were mounted with an anti-fade reagent and imaged using a fluorescence scanner with specific excitation/emission wavelengths for DAPI, FITC, CY3, and CY5. The list of antibodies used is provided in Supplementary Table 1.

12. qRT-PCR

Total RNA was isolated from cells using TRIzol reagent (Sigma-Aldrich), following the manufacturer's protocol. The extracted RNA was then reverse-transcribed into cDNA using a reverse transcription kit from TOYOBO. Quantitative real-time PCR (qRT-PCR) was carried out using the SYBR Green system (Applied Biosystems). GAPDH served as the internal control gene for normalization of gene expression. Relative expression levels of target genes were determined using the $2^{-\Delta\Delta Ct}$ method. The sequences of all primers used are provided in Supplementary Table 4.

Supplementary Table 1.

Immunohistochemistry (IHC) and Immunofluorescence antibodies

**Antibody**	**Provider**	**Host**	**Catelog No.**
Claudin-7	Abcam	Rabbit	ab27487
CD66B	Abcam	Rabbit	ab207718
CD8	Abcam	Rabbit	ab237709
Ki67	Abcam	Rabbit	ab15580

Supplementary Table 2 Flow cytometry (FCAS) antibodies and reagents

Antibody	Name	Provider	Catelog No.
CD45	Brilliant Violet 605™ anti-mouse CD45	Biolegend	Cat#103139
CD11b	PE/Cyanine7 anti-mouse/human CD11b	Biolegend	Cat#101215
Ly6G	FITC anti-mouse Ly-6G	Biolegend	Cat#127605
F4/80	Brilliant Violet 421™ anti-mouse F4/80	Biolegend	Cat#123131
CD86	APC anti-mouse CD86	Biolegend	Cat#159215
CD206	PE anti-mouse CD206 (MMR)	Biolegend	Cat#141706
CD4	Alexa Fluor® 488 anti-mouse CD4	Biolegend	Cat#100529
CD3	PE/Cyanine7 anti-mouse CD3	Biolegend	Cat#100220
CD8	PE anti-mouse CD8a	Biolegend	Cat#162304
IFN-γ	Brilliant Violet 421™ anti-mouse IFN-γ	Biolegend	Cat#505829
TNF-α	PE/Dazzle™ 594 anti-mouse TNF-α	Biolegend	Cat#506346
Zombie	Zombie NIR™ Fixable Viability Kit Red Laser	Biolegend	Cat#423105
CD16/32	Purified anti-mouse CD16/32 Antibody	Biolegend	Cat#101301
PD-L1	PerCP/Cyanine5.5 anti-mouse PDL1	Biolegend	Cat# 124334

Supplementary Table 3 Western blot antibodies

Antibody	Provider	Host	Catelog No.
NF-kB p65 Antibody	Affinity	Rabbit	AF5006
Phospho-NF-kB p65	Affinity	Rabbit	AF2006
beta-Actin Antibody	Affinity	Mouse	T0022
PDL1	Cell Signaling Technology	Rabbit	#13684
Pankla	ABclonal	Rabbit	A23004
Histone H3	Cell Signaling Technology	Rabbit	9715S
GAPDH	Cell Signaling Technology	Mouse	97166
H3K18la	ABclonal	Rabbit	A18807

Supplementary Table 4 qPCR Primer

Gene name	Forward Sequence	Reverse Sequence
CXCL1 (mouse)	TCCAGAGCTTGAAGGTGTTGCC	AACCAAGGGAGCTTCAGGGTCA
CCL7 (mouse)	CAGAAGGATCACCAGTAGTCGG	ATAGCCTCCTCGACCCACTTCT
CCL5 (mouse)	CCTGCTGCTTTGCCTACCTCTC	ACACACTTGGCGGTTCCTTCGA
CCL2 (mouse)	GCTACAAGAGGATCACCAGCAG	GTCTGGACCCATTCCTTCTTGG
CXCL10 (mouse)	ATCATCCCTGCGAGCCTATCCT	GACCTTTTTTGGCTAAACGCTTTC
HMOX1 (mouse)	CACTCTGGAGATGACACCTGAG	GTGTTCCTCTGTCAGCATCACC
PFK1 (mouse)	CTGTTCGCTCTACCGTGAGGAT	TTGGAACCACCTTGACCAGTCC
HSP90AA1 (mouse)	GCTTTCAGAGCTGTTGCGGTAC	AAAGGCGGAGTTAGCAACCTGG
NOS2 (mouse)	GAGACAGGGAAGTCTGAAGCAC	CCAGCAGTAGTTGCTCCTCTTC
NOX2 (mouse)	TGGCGATCTCAGCAAAAGGTGG	GTACTGTCCCACCTCCATCTTG
PDL2 (mouse)	CTGGGACTACAAGTACCTGACG	CTCTAGCCTGGCAGGTAAGCTG
PTGS2 (mouse)	GCGACATACTCAAGCAGGAGCA	AGTGGTAACCGCTCAGGTGTTG
ADAM17 (mouse)	TGTGAGCGGTGACCACGAGAAT	TTCATCCACCCTGGAGTTGCCA
LGALS9 (mouse)	CTGGAATCCCTCCTGTGGTGTA	CCTCGTAGCATCTGGCAAGACA
PTGER2 (mouse)	GAGAGAGGACTTCGATGGCAGA	GGAAGAGGTTTCATCCATGTAGG
SLC7A2 (mouse)	GTGTGCCTTGTATTACTCCTGGC	CCACCATGACAAAGAGAAGGACC
TGFB2 (mouse)	TTGTTGCCCTCCTACAGACTGG	GTAAAGAGGGCGAAGGCAGCAA
ARG1 (mouse)	CATTGGCTTGCGAGACGTAGAC	GCTGAAGGTCTCTTCCATCACC
CCL5 (mouse)	CCTGCTGCTTTGCCTACCTCTC	ACACACTTGGCGGTTCCTTCGA

CD47 (mouse)	GGTGGGAAACTACACTTGCGAAG	CTCCTCGTAAGAACAGGCTGATC
ALOX5 (mouse)	TCTTCCTGGCACGACTTTGCTG	GCAGCCATTCAGGAACTGGTAG
NLRP3 (mouse)	TCACAACTCGCCCAAGGAGGAA	AAGAGACCACGGCAGAAGCTAG
SPP1 (mouse)	GCTTGGCTTATGGACTGAGGTC	CCTTAGACTCACCGCTCTTCATG
HMOX1 (mouse)	CACTCTGGAGATGACACCTGAG	GTGTTCCTCTGTCAGCATCACC
CXCL1(human)	AGCTTGCCTCAATCCTGCATCC	TCCTTCAGGAACAGCCACCAGT
CCL7(human)	ACAGAAGGACCACCAGTAGCCA	GGTGCTTCATAAAGTCCTGGACC
CCL5(human)	CCTGCTGCTTTGCCTACATTGC	ACACACTTGGCGGTTCTTTCGG
CCL2(human)	AGAATCACCAGCAGCAAGTGTCC	TCCTGAACCCACTTCTGCTTGG
CXCL10(human)	GGTGAGAAGAGATGTCTGAATCC	GTCCATCCTTGGAAGCACTGCA

Supplementary Figure

Supplementary Figure 1 Supplement presents the results of single-cell data analysis, further elucidating gene expression markers and functional annotations of different cell types within the tumor microenvironment.

(A) The UMAP plot displays the expression of key cell type markers within the dataset, including EPCAM (epithelial cells), VWF (endothelial cells), PDGFRA (fibroblasts), ACTA2 (smooth muscle cells), CD163 (macrophages), MS4A2 (mast cells), CSF3R (granulocytes), CD3E (T cells), GNLY (natural killer cells), MS4A1 (B cells), MZB1 (plasma cells), and MKI67 (proliferating cells). These markers aid in distinguishing and localizing cell populations within the tumor microenvironment. **(B)** The dot plot shows the distribution and expression levels of these marker genes across different cell types, with the size of each dot indicating the percentage of cells expressing the gene and color intensity representing the average expression level. This plot categorizes major cell types as epithelial, endothelial, smooth muscle, fibroblast, myeloid, mast, neutrophil, T cell, NK cell, B cell, plasma, and proliferating cells. **(C)** UMAP dimensionality reduction was used to visualize the subclustering of epithelial cells based on single-cell transcriptomic data. A total of 13,107 epithelial cells were identified and further classified into 14 distinct subclusters (Epithelial-1 to Epithelial-14) according to their gene expression profiles. Each subcluster is color-coded, with the number of cells in each group indicated in parentheses. **(D)** Copy number variation (CNV) scores were calculated for each epithelial subcluster using the inferCNV tool to assess genomic instability. The distribution of CNV scores across the different CNV clusters (Epi-1 to Epi-14) is visualized using box plots.

Supplementary Figure 2. Flow cytometry analysis of macrophage and neutrophil populations in MC38 and CMT93 tumors.

(A) Gating strategy for identifying various cell populations, including neutrophils ($Ly6G^{+}CD11b^{+}$) and macrophages ($F4/80^{+}$), and further differentiating M1 and M2 macrophages ($CD86^{+}$ and $CD206^{+}$, respectively). **(B)** MC38 tumors showing the percentage of $CD11b^{+}CD45^{+}$ cells, macrophages, M1 and M2 macrophages in control (Ctrl) and CLDN7 overexpression (Cldn7oe) groups. **(C)** CMT93 tumors showing similar analysis for $CD11b^{+}CD45^{+}$ cells, macrophages, M1 and M2 macrophages in shNC and shCldn7 groups. Significant differences are marked by asterisks (***) and non-significant differences are marked as ns.

Supplementary Figure 3. Flow cytometry analysis of T cell populations in MC38 and CMT93 tumors.

(A) Gating strategy for identifying $CD4^+$ and $CD8^+$ T cells, and further differentiating subsets based on IFN-γ, TNF-α, and FOXP3 expression. **(B)** MC38 tumors showing the percentage of $CD4^+/CD3^+$ T cells, $FOXP3^+$ $CD4^+$ T cells, IFN-γ^+ $CD4^+$ T cells, and TNF-α^+ $CD4^+$ T cells in the Ctrl and *Cldn7*oe groups.
(C) CMT93 tumors showing similar analysis for $CD4^+/CD3^+$ T cells, $FOXP3^+$ $CD4^+$ T cells, IFN-γ^+ $CD4^+$ T cells, and TNF-α^+ $CD4^+$ T cells in the shNC and sh*Cldn7* groups.

Supplementary Figure 4. Apoptosis analysis of neutrophils (Neu) cultured in conditioned media from tumor cells with different *Cldn7* expression levels.

(A) Apoptosis analysis of neutrophils cultured in conditioned media from tumor cells with different CLDN7 expression levels. Flow cytometry results show no significant difference in neutrophil apoptosis rates among groups with control, CLDN7 overexpression, or knockdown in MC38, CMT93, and HCT116 cells.

Supplementary Figure 5. Gene pathway analysis of MC38 cells with Cldn7 overexpression (mc38oe) compared to control (mc38nc).

(A) KEGG pathway analysis showing the distribution of genes involved in various biological processes. The pathways are grouped into categories such as metabolism, environmental information processing, cellular processes, organismal systems, and human diseases. Notably, the immune system and infectious disease pathways are significantly enriched. **(B)** Reactome pathway analysis at Level 1 highlighting the major biological processes, including immune system, metabolism, and gene expression. A substantial number of genes are involved in immune response and signaling.

Supplementary Figure 6. (A) KEGG enrichment analysis of gene pathways. The bubble plot shows the enrichment of different pathways based on the gene expression data. Each bubble represents a specific pathway, with the size of the bubble indicating the number of genes involved in that pathway and the color representing the statistical significance (Padjust). Pathways such as TNF signaling, NF-kappa B signaling, and IL-17 signaling are significantly enriched, indicating their potential involvement in the biological processes under study.

Supplementary Figure 7. *Cldn7* overexpression enhances the immunotherapeutic efficacy of PD-1 blockade in MC38 and CT26 tumor models.

(A–C) Flow cytometry analysis of immune cell populations in MC38 tumors from mice treated with control (Ctrl), anti–PD-1 antibody (Anti–PD-1), Cldn7 overexpression (Cldn7oe), or combination therapy (Comb.). **(A)** Proportional changes in myeloid cells and their subsets within the tumor microenvironment. **(B)** PD-1 expression levels on $CD3^+$ $CD8^+$ T cells, quantified by mean fluorescence intensity (MFI). **(C)** TIM3 expression levels on $CD3^+$ $CD8^+$ T cells (MFI). Combined treatment with Cldn7 overexpression and PD-1 blockade significantly reduced the expression of PD-1 and TIM3, indicating alleviated T cell exhaustion and enhanced T cell activation. **(D–F)** Parallel analyses in CT26 tumor-bearing mice subjected to the same

treatment regimens. **(D)** Composition of myeloid cells and their subsets across treatment groups. **(E)** Quantification of PD-1 expression on CD3⁺ CD8⁺ T cells. (F) TIM3 expression levels on CD3⁺ CD8⁺ T cells. The combination therapy markedly decreased PD-1 and TIM3 expression, further supporting that Cldn7 overexpression potentiates the efficacy of PD-1 immune checkpoint inhibition by improving T cell functionality.

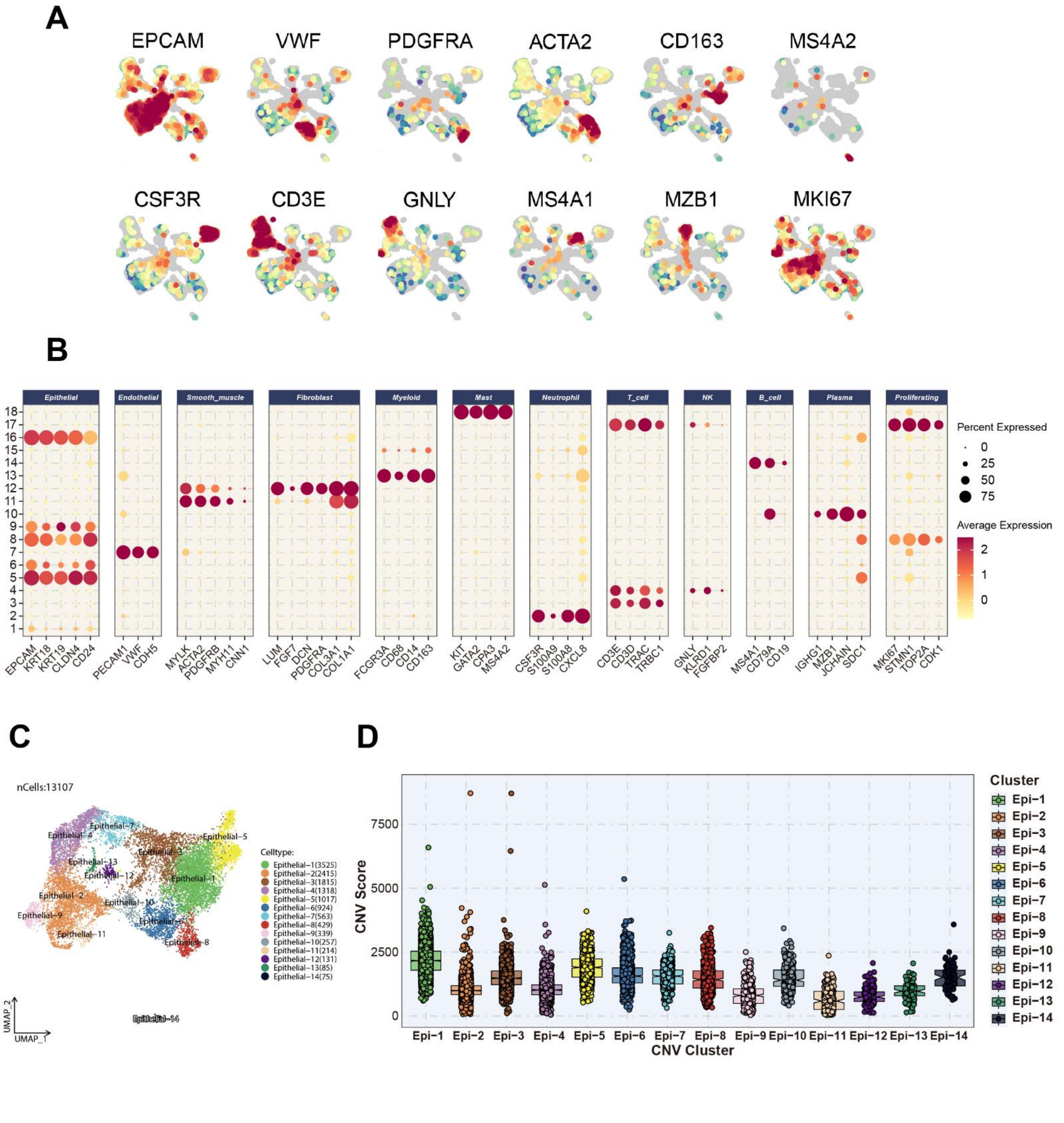

Figure S1

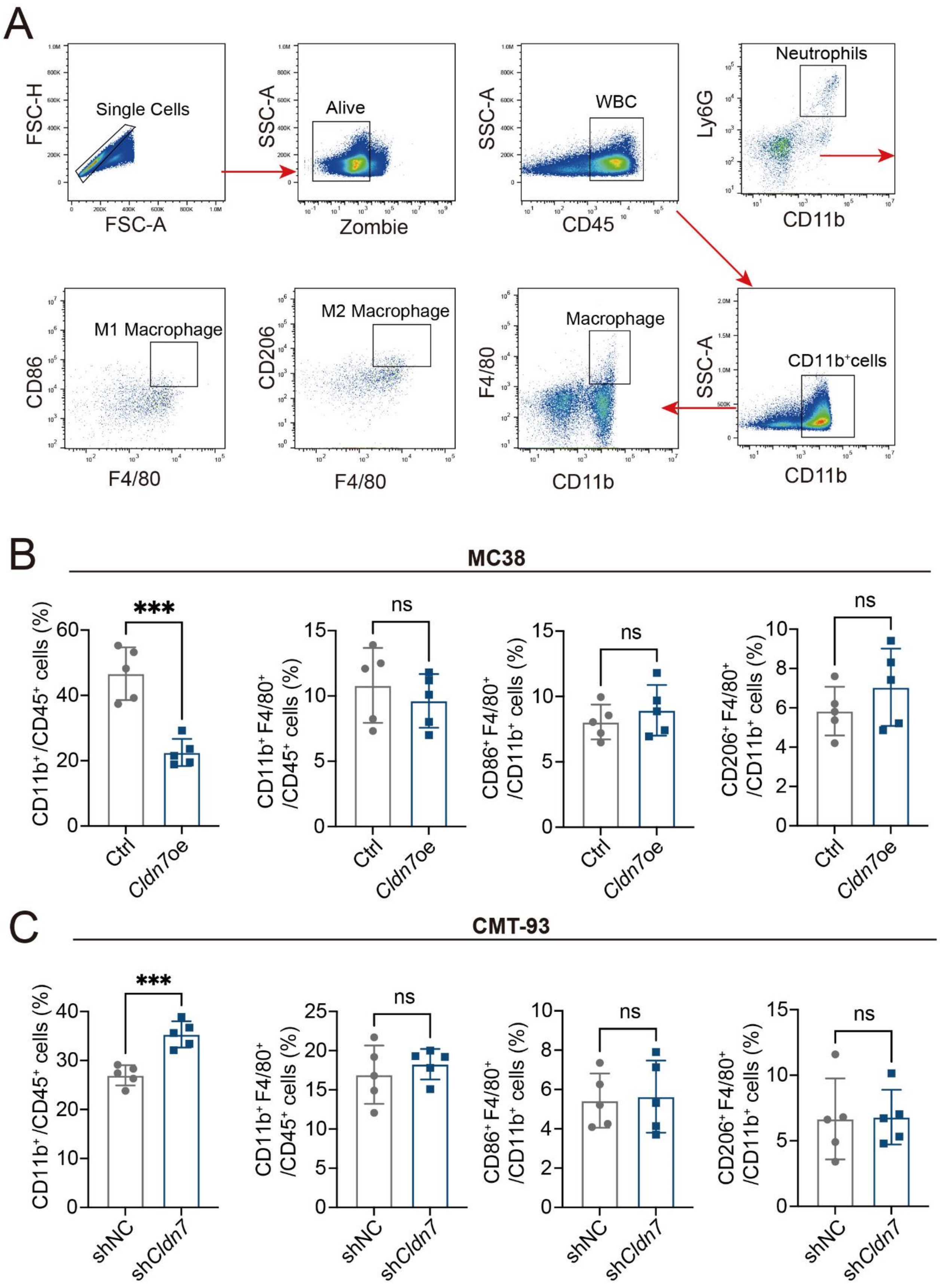
A
Single Cells
FSC-H
FSC-A
Alive
SSC-A
Zombie
WBC
SSC-A
CD45
Neutrophils
Ly6G
CD11b
M1 Macrophage
CD86
F4/80
M2 Macrophage
CD206
F4/80
Macrophage
F4/80
CD11b
CD11b⁺cells
SSC-A
CD11b
B
MC38
***
ns
ns
ns
CD11b⁺/CD45⁺ cells (%)
CD11b⁺ F4/80⁺ /CD45⁺ cells (%)
CD86⁺ F4/80⁺ /CD11b⁺ cells (%)
CD206⁺ F4/80⁺ /CD11b⁺ cells (%)
Ctrl
Cldn7oe
C
CMT-93
***
ns
ns
ns
shNC
shCldn7

Figure S2

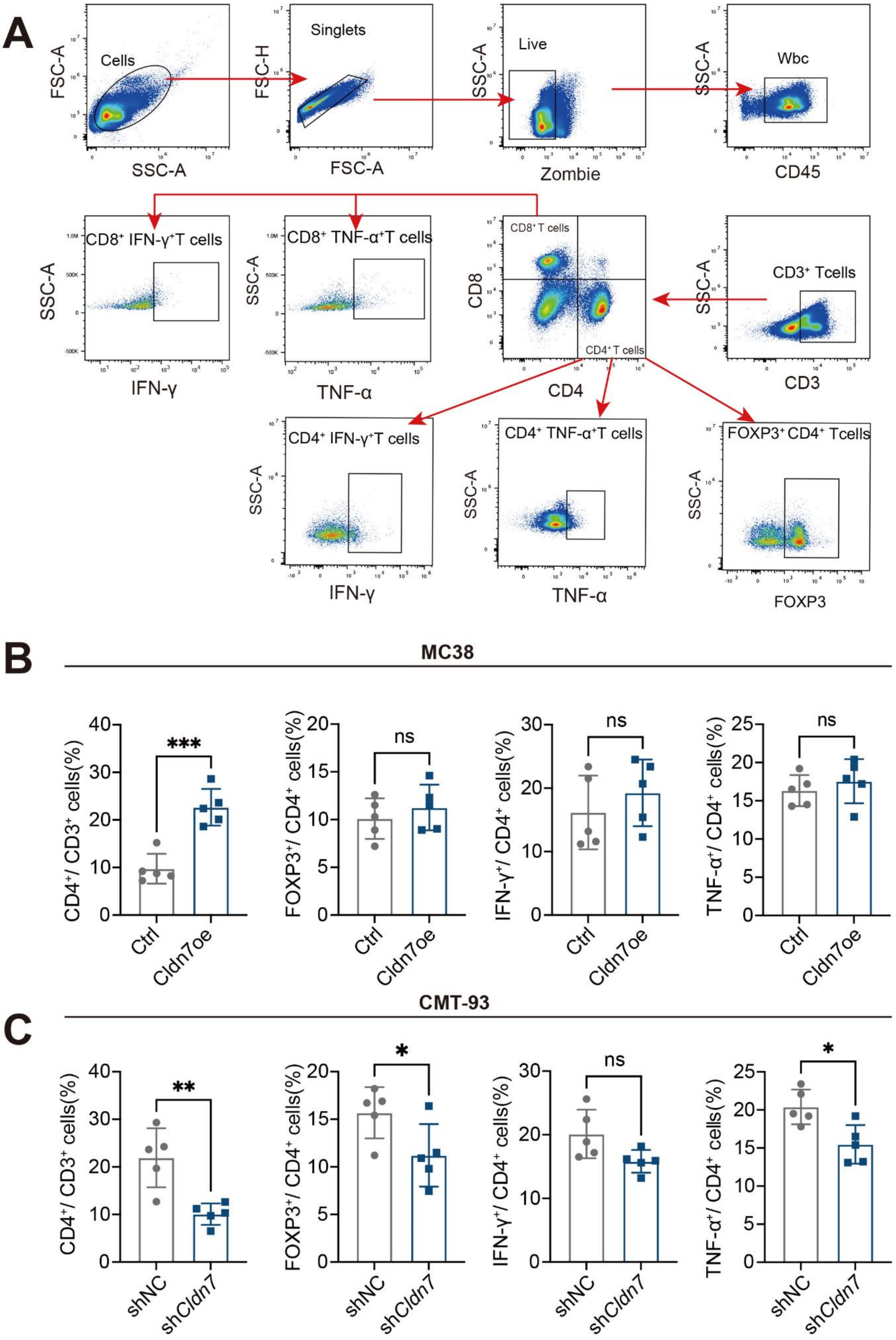
A
Cells
FSC-A
SSC-A
Singlets
FSC-H
FSC-A
Live
SSC-A
Zombie
Wbc
SSC-A
CD45
CD8⁺ IFN-γ⁺T cells
SSC-A
IFN-γ
CD8⁺ TNF-α⁺T cells
SSC-A
TNF-α
CD8⁺ T cells
CD4⁺ T cells
CD8
CD4
CD3⁺ Tcells
SSC-A
CD3
CD4⁺ IFN-γ⁺T cells
SSC-A
IFN-γ
CD4⁺ TNF-α⁺T cells
SSC-A
TNF-α
FOXP3⁺ CD4⁺ Tcells
SSC-A
FOXP3
B
MC38
CD4⁺/ CD3⁺ cells(%)
***
FOXP3⁺/ CD4⁺ cells(%)
ns
IFN-γ⁺/ CD4⁺ cells(%)
ns
TNF-α⁺/ CD4⁺ cells(%)
ns
Ctrl
Cldn7oe
C
CMT-93
CD4⁺/ CD3⁺ cells(%)
**
FOXP3⁺/ CD4⁺ cells(%)
*
IFN-γ⁺/ CD4⁺ cells(%)
ns
TNF-α⁺/ CD4⁺ cells(%)
*
shNC
shCldn7

Figure S3

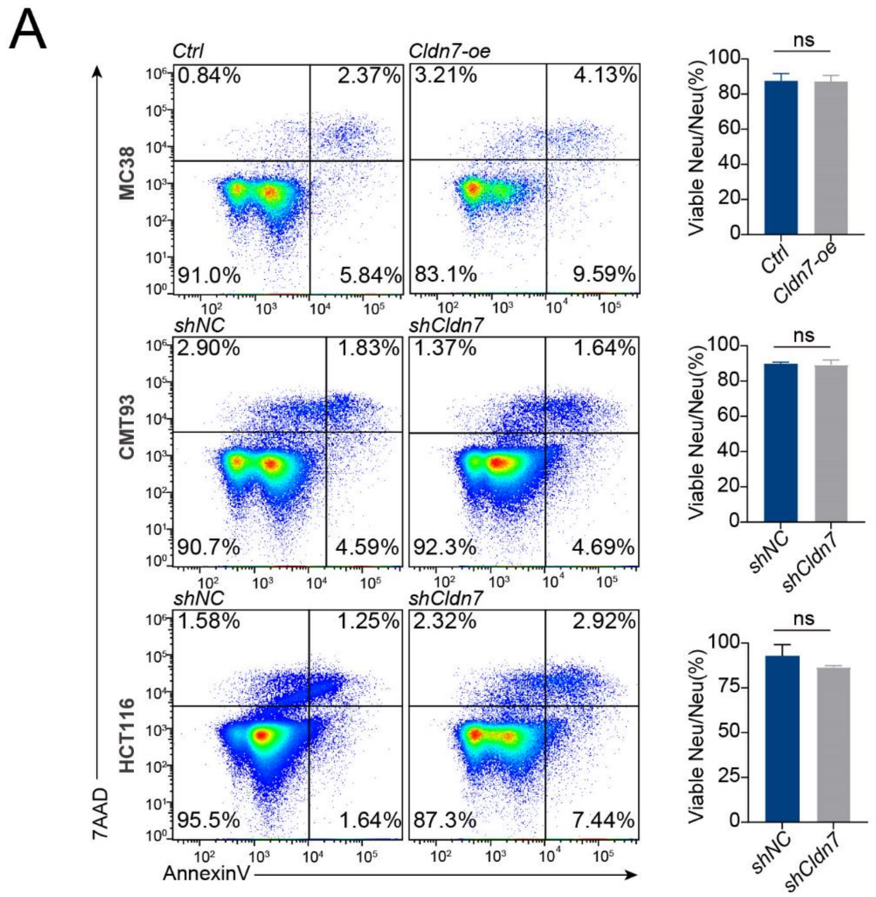
A
Ctrl
Cldn7-oe
MC38
0.84%
2.37%
91.0%
5.84%
3.21%
4.13%
83.1%
9.59%
shNC
shCldn7
CMT93
2.90%
1.83%
90.7%
4.59%
1.37%
1.64%
92.3%
4.69%
HCT116
1.58%
1.25%
95.5%
1.64%
2.32%
2.92%
87.3%
7.44%
7AAD
AnnexinV
Viable Neu/Neu(%)
ns

Figure S4

A

B

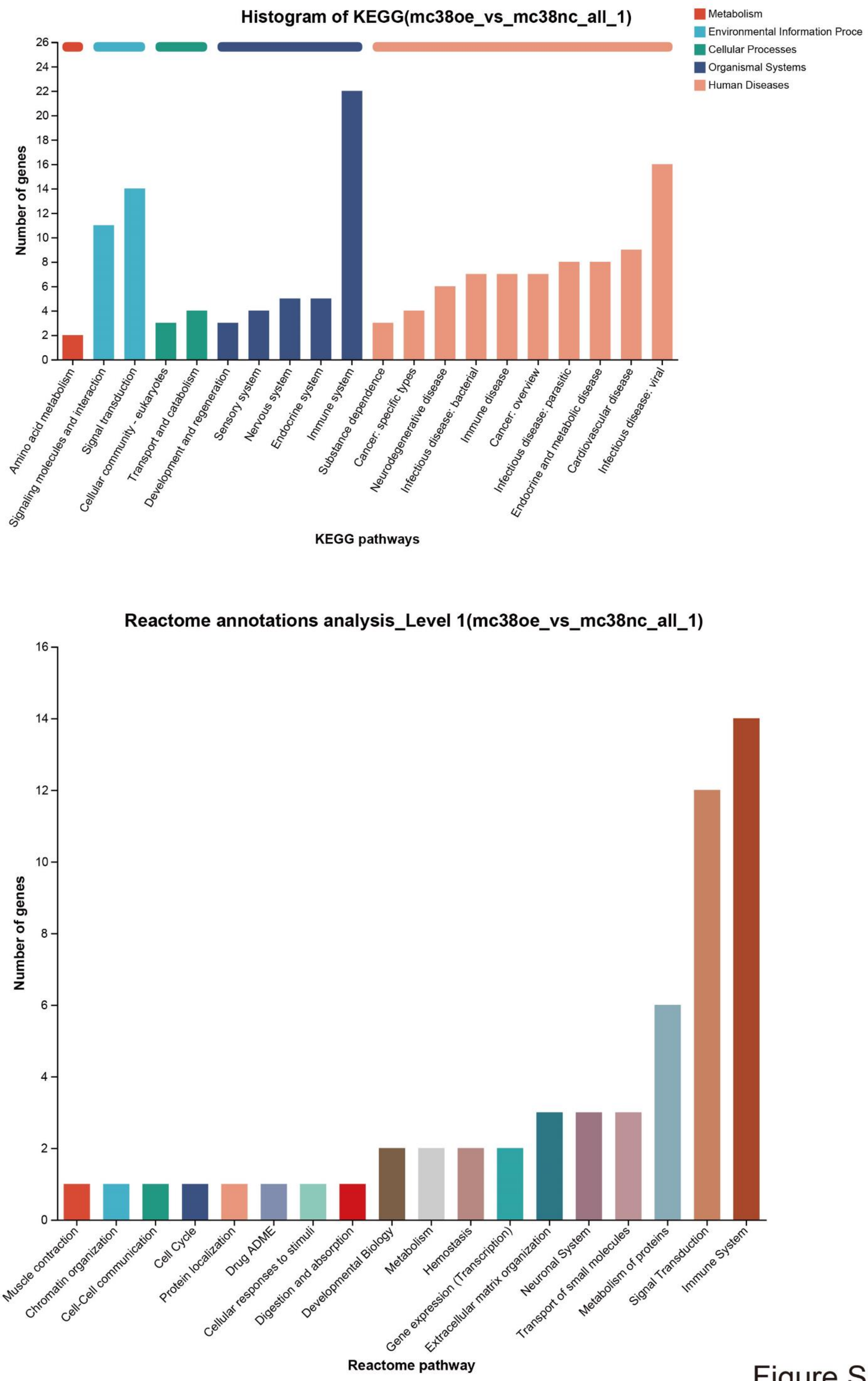
Histogram of KEGG(mc38oe_vs_mc38nc_all_1)
Metabolism
Environmental Information Proce
Cellular Processes
Organismal Systems
Human Diseases
Number of genes
0
2
4
6
8
10
12
14
16
18
20
22
24
26
Amino acid metabolism
Signaling molecules and interaction
Signal transduction
Cellular community - eukaryotes
Transport and catabolism
Development and regeneration
Sensory system
Nervous system
Endocrine system
Immune system
Substance dependence
Cancer: specific types
Neurodegenerative disease
Infectious disease: bacterial
Immune disease
Cancer: overview
Infectious disease: parasitic
Endocrine and metabolic disease
Cardiovascular disease
Infectious disease: viral
KEGG pathways
Reactome annotations analysis_Level 1(mc38oe_vs_mc38nc_all_1)
Number of genes
0
2
4
6
8
10
12
14
16
Muscle contraction
Chromatin organization
Cell-Cell communication
Cell Cycle
Protein localization
Drug ADME
Cellular responses to stimuli
Digestion and absorption
Developmental Biology
Metabolism
Hemostasis
Gene expression (Transcription)
Extracellular matrix organization
Neuronal System
Transport of small molecules
Metabolism of proteins
Signal Transduction
Immune System
Reactome pathway

Figure S5

A

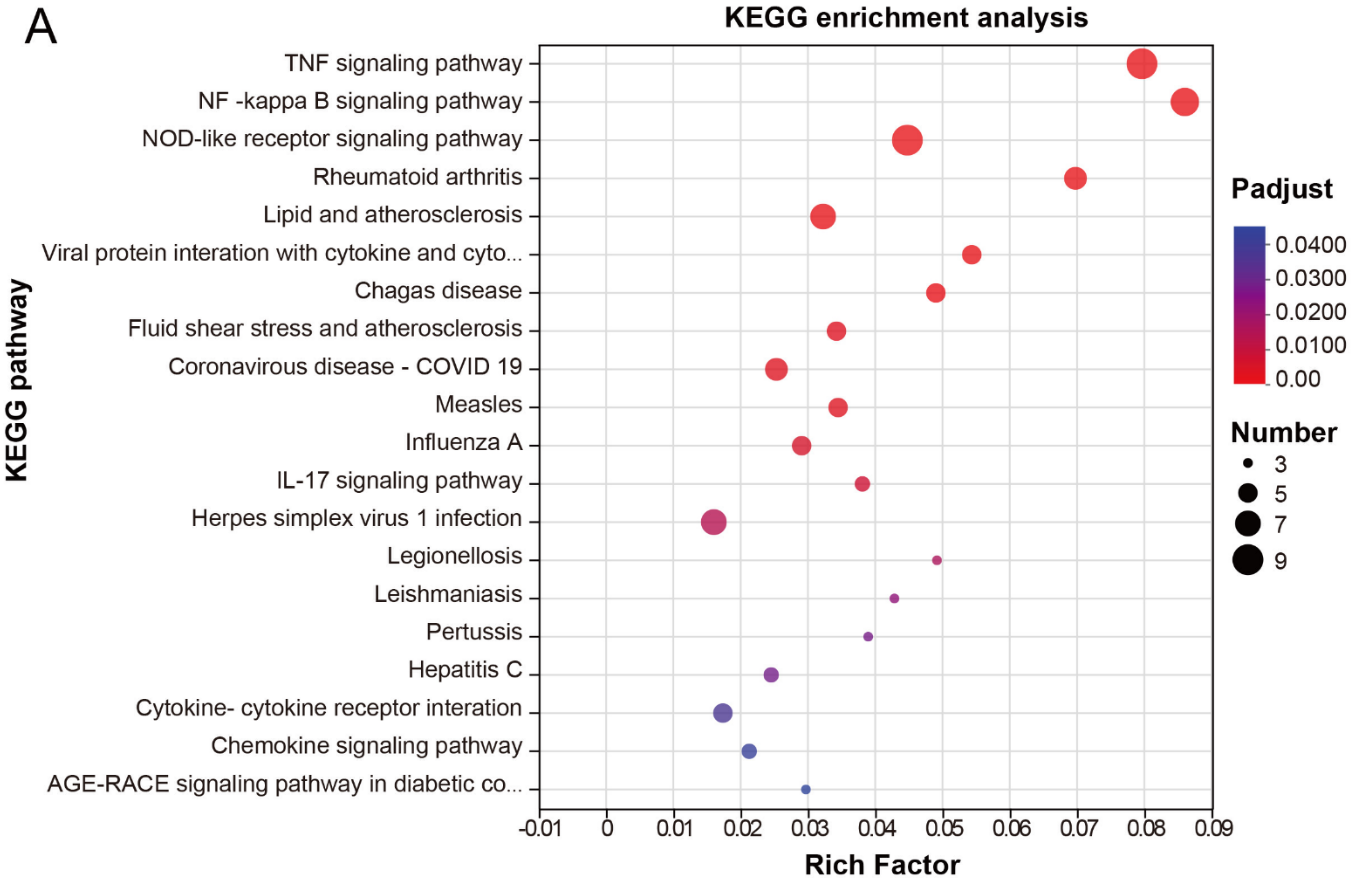

Figure S6

192

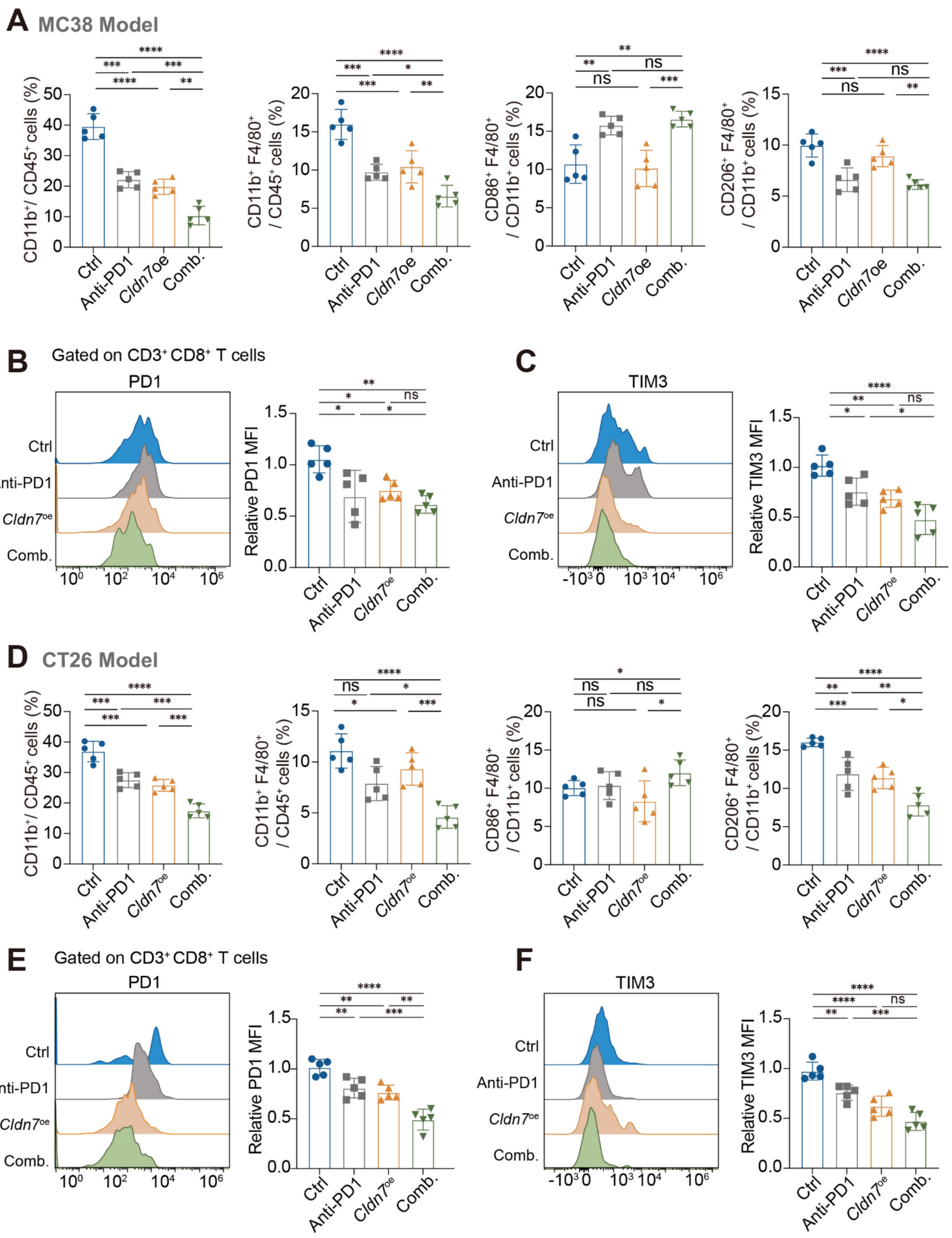

A MC38 Model
B Gated on CD3+ CD8+ T cells
PD1
TIM3
C
D CT26 Model
E Gated on CD3+ CD8+ T cells
F
Ctrl
Anti-PD1
Cldn7oe
Comb.
CD11b+/ CD45+ cells (%)
CD11b+ F4/80+ / CD45+ cells (%)
CD86+ F4/80+ / CD11b+ cells (%)
CD206+ F4/80+ / CD11b+ cells (%)
Relative PD1 MFI
Relative TIM3 MFI

Figure S7

Reference:

1. Hao Y, Hao S, Andersen-Nissen E, Mauck WM, 3rd, Zheng S, Butler A, et al. Integrated analysis of multimodal single-cell data. Cell. 2021;184(13):3573-87 e29.
2. McGinnis CS, Murrow LM, Gartner ZJ. DoubletFinder: Doublet Detection in Single-Cell RNA Sequencing Data Using Artificial Nearest Neighbors. Cell Syst. 2019;8(4):329-37 e4.
3. Yang S, Corbett SE, Koga Y, Wang Z, Johnson WE, Yajima M, et al. Decontamination of ambient RNA in single-cell RNA-seq with DecontX. Genome Biol. 2020;21(1):57.
4. Korsunsky I, Millard N, Fan J, Slowikowski K, Zhang F, Wei K, et al. Fast, sensitive and accurate integration of single-cell data with Harmony. Nat Methods. 2019;16(12):1289-96.
5. Zhou Y, Bian S, Zhou X, Cui Y, Wang W, Wen L, et al. Single-Cell Multiomics Sequencing Reveals Prevalent Genomic Alterations in Tumor Stromal Cells of Human Colorectal Cancer. Cancer Cell. 2020;38(6):818-28.e5.
6. Chen Y, Wang D, Li Y, Qi L, Si W, Bo Y, et al. Spatiotemporal single-cell analysis decodes cellular dynamics underlying different responses to immunotherapy in colorectal cancer. Cancer Cell. 2024;42(7):1268-85.e7.
7. Wang L, Liu Y, Dai Y, Tang X, Yin T, Wang C, et al. Single-cell RNA-seq analysis reveals BHLHE40-driven pro-tumour neutrophils with hyperactivated glycolysis in pancreatic tumour microenvironment. Gut. 2023;72(5):958-71.
8. Chu X, Li X, Zhang Y, Dang G, Miao Y, Xu W, et al. Integrative single-cell analysis of human colorectal cancer reveals patient stratification with distinct immune evasion mechanisms. Nat Cancer. 2024;5(9):1409-26.
9. Jin S, Guerrero-Juarez CF, Zhang L, Chang I, Ramos R, Kuan CH, et al. Inference and analysis of cell-cell communication using CellChat. Nat Commun. 2021;12(1):1088.
