# Supplementary material for: Claudin-7 deficiency induces metabolic reprogramming of neutrophils in the colorectal cancer microenvironment
Source: Cell Death Dis. 2025 Oct 16;16(1):728. doi: 10.1038/s41419-025-08064-3 (PMC12533015; doi:10.1038/s41419-025-08064-3)
Supplement: Supplementary file 2 — Original data for western blot [file 41419_2025_8064_MOESM2_ESM.pdf]

Original data for western blot

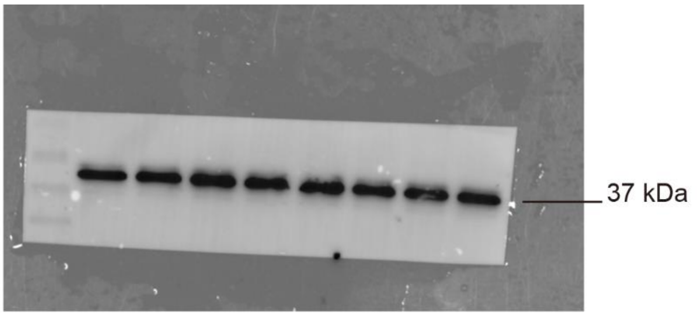

GAPDH Related to Figure 2F

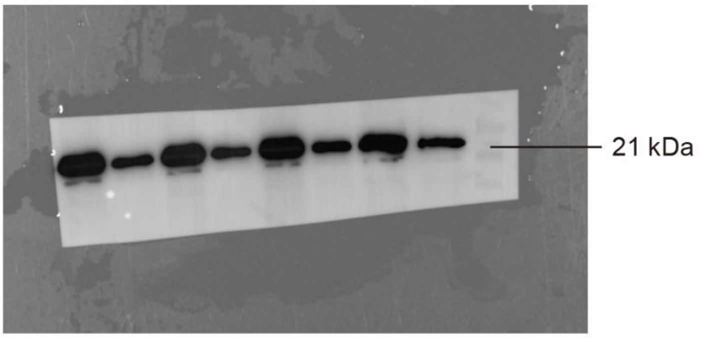

Cldn7 Related to Figure 2F

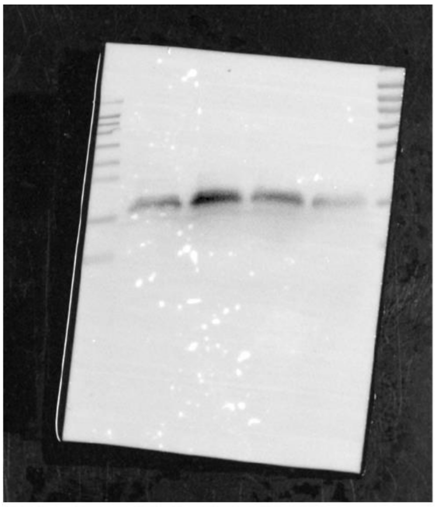

PD-L1 Related to Figure 7K

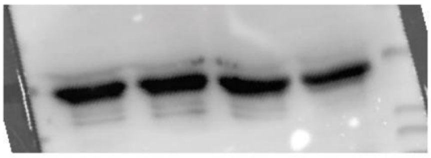

GAPDH related to Figure 7K

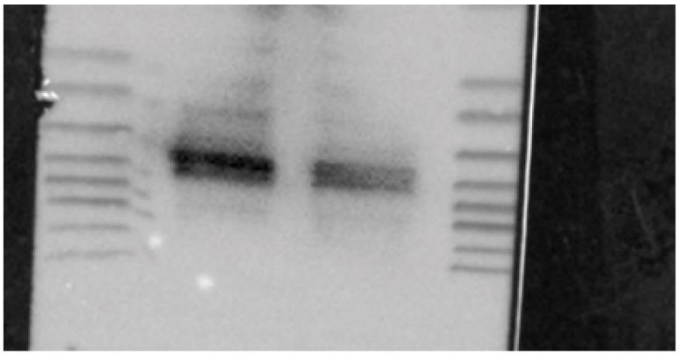

p-p65 related to Figure6A

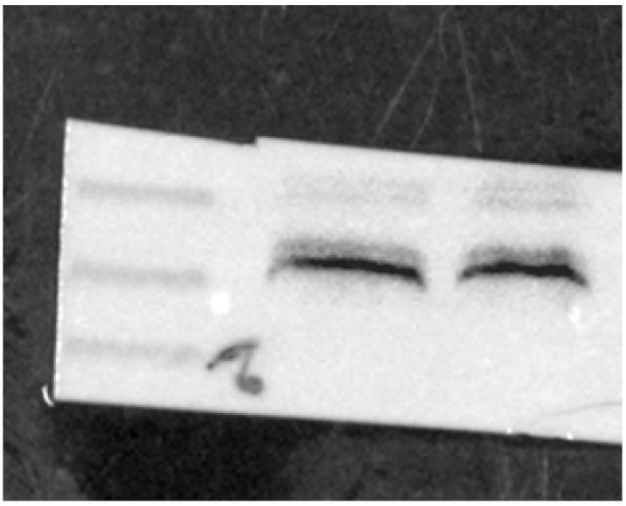

p65 related to Figure6A

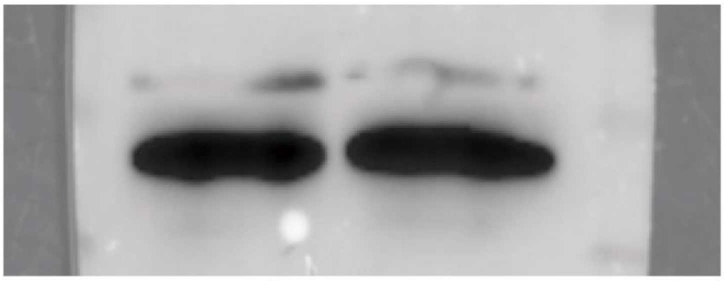

Gapdh related to Figure6A

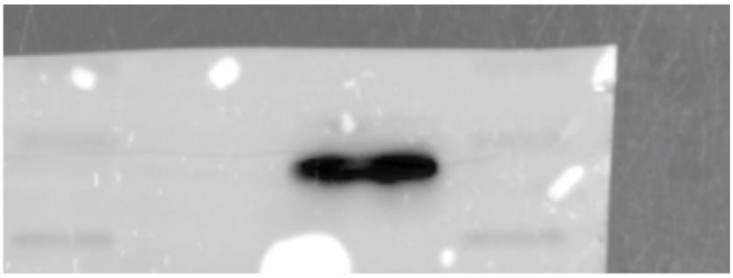

Cldn7 related to Figure6A

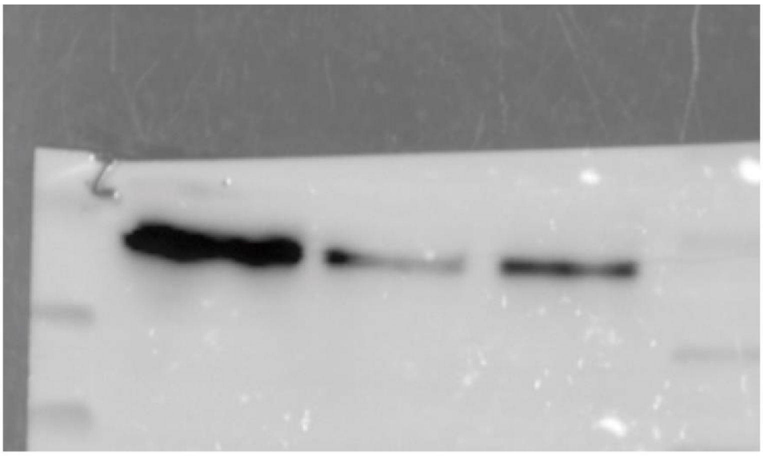

Cldn7 related to Figure 6B

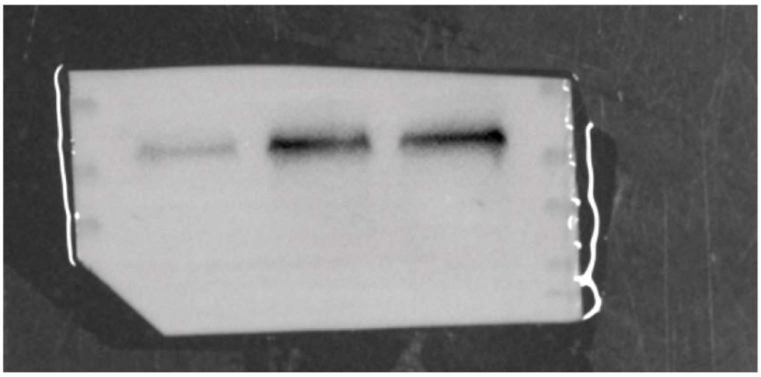

p-p65 related to Figure 6B

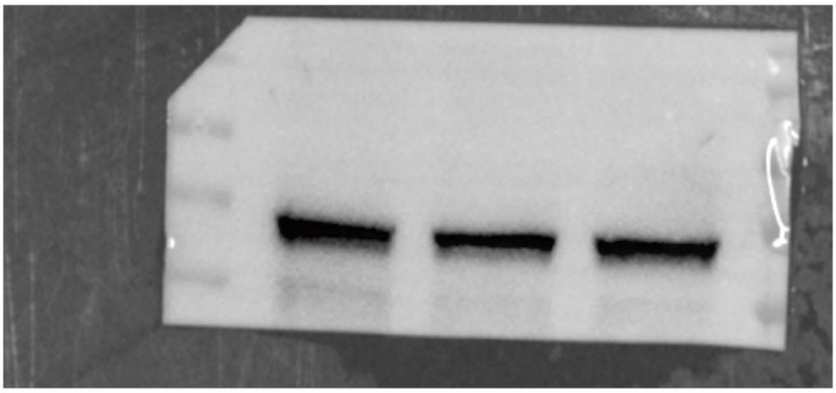

p65 related to Figure 6B

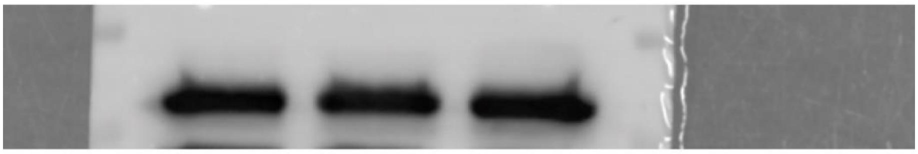

Gapdh related to Figure 6B

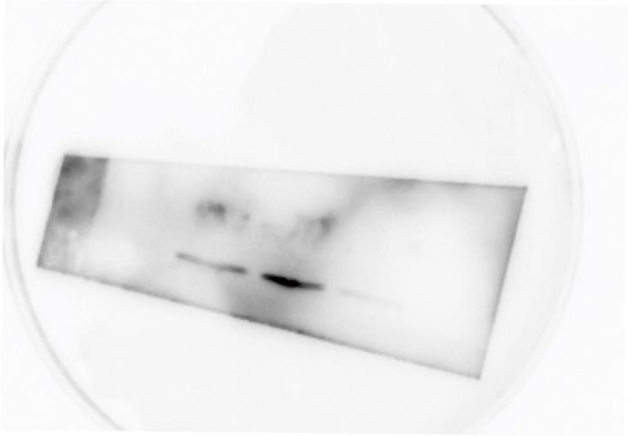

H3k18la related to Figure 7M

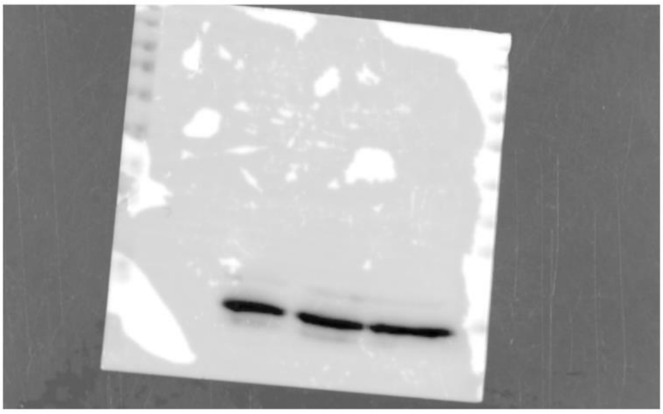

Hisotone H3 related to Figure 7M

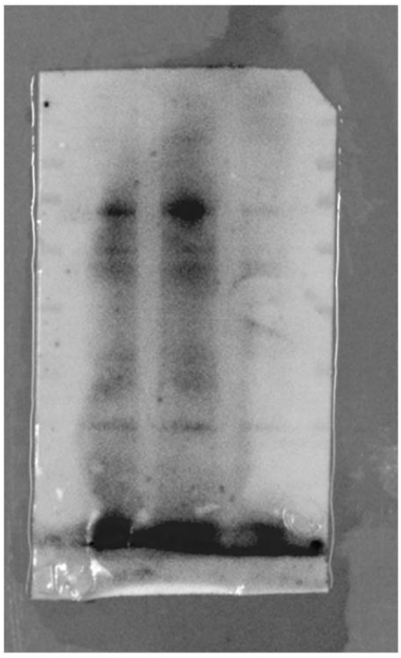

Pankla related to Figure 7M

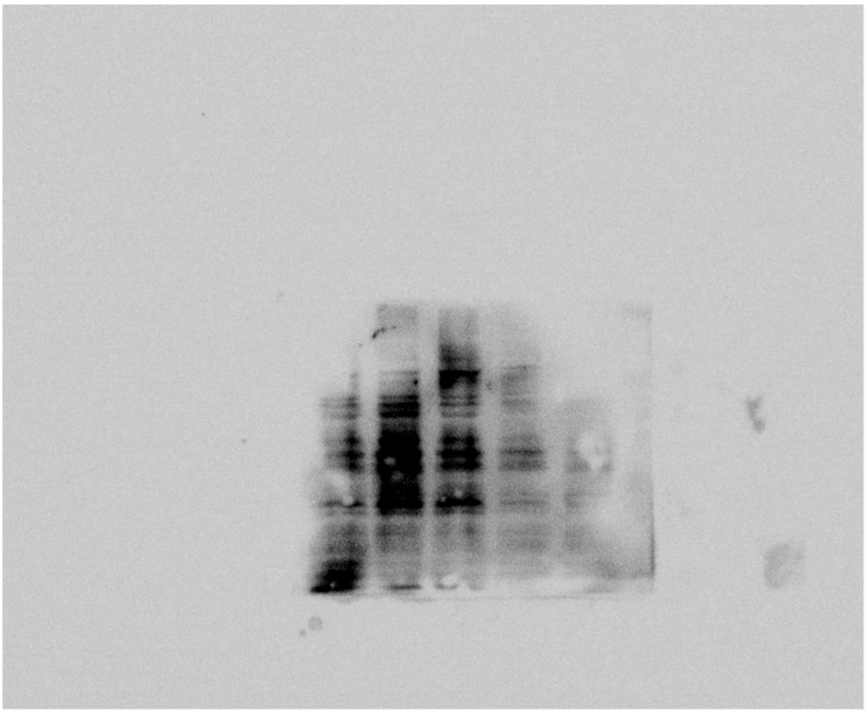

Pankla related to figure 7N

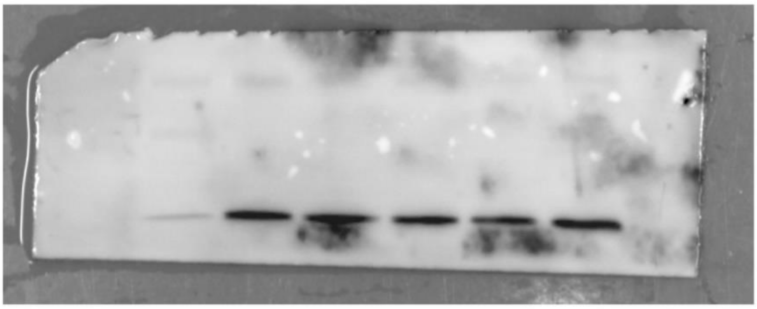

Hisotone H3 related to figure 7N

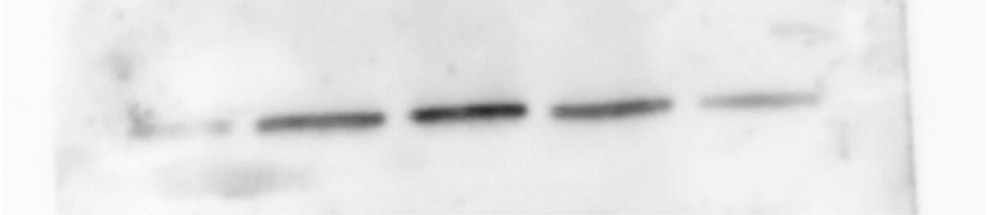

H3k18la related to figure 7N
